# Supplementary material for: A single dose of ChAdOx1 Chik vaccine induces neutralizing antibodies against four chikungunya virus lineages in a phase 1 clinical trial
Source: Nat Commun. 2021 Jul 30;12:4636. doi: 10.1038/s41467-021-24906-y (PMC8324904; doi:10.1038/s41467-021-24906-y)
Supplement: Supplementary file 1 — Supplementary Information [file 41467_2021_24906_MOESM1_ESM.pdf]

Supplementary Appendix

This appendix has been provided by the authors to give readers additional information about their work.

Table S1 | Duration of Adverse Events (AEs)

Table S2 | Laboratory AEs considered possibly, probably or definitely related with ChAdOx1 Chik.

Table S3 | Unsolicited AEs considered possibly, probably or definitely related with ChAdOx1 Chik.

Table S4 | Peptide pools used for *ex vivo* IFN-γ ELISpot and Intracellular Cytokine Staining (ICS) by flow cytometry.

Figure S1 | ICS gating strategy.

Figure S2 | Maximum $PRNT_{50}$ neutralisation titres against four CHIKV lineages.

Study Protocol | A phase I study to determine the safety and immunogenicity of the candidate Chikungunya Virus (CHIKV) vaccine ChAdOx1 Chik in healthy adult volunteers.

Table S1. Duration of AEs

AE	Median	IQR	Maximum duration
**Pain at injection site**	3.00	2-3	7.00
**Pruritus**	-	-	-
**Warmth**	1.00	1-1	3.00
**Erythema**	1.00	1-1	1.00
**Swelling**	-	-	-
**Fever**	1.00	1-1	1.00
**Feverishness**	2.00	1-3	3.00
**Arthralgia**	2.00	1-2	2.00
**Myalgia**	2.00	1-2	3.00
**Headache**	2.00	1-3	5.00
**Fatigue**	1.00	1-2	5.00
**Nausea**	1.00	1-2	2.00
**Malaise**	2.00	1-2.75	3.00
**Overall**	2.00	1-3	7.00

Table S2. Laboratory AEs considered possibly, probably or definitely related with ChAdOx1 Chik

	Id	Group	Event	Severity	Timepoint	Resolved by	Comment
**Haematology**	101014	2	Leucopenia	Mild	D2	D7	
	101014	2	Neutropenia	Mild	D2	D7	
	101018	3	Lymphopenia	Mild	D2	D7	
	101019	3	Leucopenia	Mild	D2	D7	
	101019	3	Neutropenia	Mild	D2	D7	
	101029	3	Leucopenia	Mild	D2	D7	
	101029	3	Neutropenia	Mild	D2	D7	
	101030	2	Leucopenia	Mild	D2	D7	
	101030	2	Neutropenia	Moderate	D2	D28	
	101036	2	Leucopenia	Mild	D2	D7	
	101036	2	Neutropenia	Mild	D2	D7	
**Biochemistry**	101003	1	Hyperbilirubinemia	Moderate	D2	Not resolved	Participant has known Gilbert's Sd.<br>Bilirubin=28 at D28 (baseline bilirubin=24)
	101014	2	Hypoalbuminemia	Mild	D2	Not resolved	Alb=33 at baseline; 31 at D2; 32 at D7; 31 at D28; 31 at D56.<br>Although meets toxicity grading scale criteria (mild),this was not considered to be clinically significant
	101017	3	Hyperbilirubinemia	Moderate	D2	D7	Highest Bilirubin=34. Normal ALT

Table S3. Unsolicited AEs considered possibly, probably or definitely related with ChAdOx1 Chik

Subject ID	MEDDRA PT	MEDDRA PT CODE	Day of Onset	Duration (days)	Max. Severity
00101012	Dizziness	10013573	0	1	1
00101016	Muscle spasms	10028334	4	1	1
00101006	Cough	10011224	4	2	1
00101018	Oropharyngeal pain	10068319	0	2	1
00101018	Cough	10011224	0	8	1
00101017	Chills	10008531	1	0	1
00101019	Chills	10008531	0	1	2
00101027	Chills	10008531	0	2	3
00101027	Back pain	10003988	0	2	2
00101027	Muscular weakness	10028372	1	1	2
00101027	Sleep disorder	10040984	2	1	3
00101029	Hypoaesthesia	10020937	0	3	2
00101037	Hunger	10020466	0	1	1
00101031	Coordination abnormal	10010947	0	1	2
00101030	Back pain	10003988	0	2	3
00101030	Nasal congestion	10028735	2	1	1
00101041	Rhinorrhoea	10039101	3	3	2
00101011	Somnolence	10041349	0	1	1
00101027	Hypoaesthesia	10020937	0	1	1

Table S4. Peptide pools used for *ex vivo* IFN-γ ELISpot and Intracellular cytokine Staining (ICS) by flow cytometry

ELISPOT Pool preparation

Peptide Pool	Peptide Name	Stock Conc. (μg/mL)	Pool Conc. Required (μg/mL)	Dilution Factor	Pool Volume (μL)	Volume of Peptide to Add (μL)	R10 Volume (μL)
Capsid 1	MEFIPTQTFYNRRYQPRPWT	100000	20	5000	30000	6	
	NRRYQPRPWTPRPTIQVIRP	100000	20	5000	30000	6	
	PRPTIQVIRPRPRPQRQAGQ	100000	20	5000	30000	6	
	RPRPQRQAGQLAQLISAVNK	100000	20	5000	30000	6	
	LAQLISAVNKLTMRAVPQQK	100000	20	5000	30000	6	
	LTMRAVPQQKPRKNRKNKKQ	100000	20	5000	30000	6	
	PRKNRKNKKQKQKQQAPQND	100000	20	5000	30000	6	
	KQKQQAPQNDPKQKKQPPQK	100000	20	5000	30000	6	29952
Capsid 2	PKQKKQPPQKKPAQKKKKPG	100000	20	5000	30000	6	
	KPAQKKKKPGRRERMCMKIE	100000	20	5000	30000	6	
	RRERMCMKIENDCIFEVKHE	100000	20	5000	30000	6	
	NDCIFEVKHEGKVMGYACLV	100000	20	5000	30000	6	
	GKVMGYACLVGDKVMKPAHV	100000	20	5000	30000	6	
	GDKVMKPAHVKGTIDNADLA	100000	20	5000	30000	6	
	KGTIDNADLAKLAFKRSSKY	100000	20	5000	30000	6	
	KLAFKRSSKYDLECAQIPVH	100000	20	5000	30000	6	
	DLECAQIPVHMKSDASKFTH	100000	20	5000	30000	6	29946
Capsid 3	MKSDASKFTHEKPEGYYNWH	100000	20	5000	30000	6	
	EKPEGYYNWHHGAVQYSGGR	100000	20	5000	30000	6	
	HGAVQYSGGRFTIPTGAGKP	100000	20	5000	30000	6	
	FTIPTGAGKPGDSGRPIFDN	100000	20	5000	30000	6	
	GDSGRPIFDNKGRVVAIVLG	100000	20	5000	30000	6	
	KGRVVAIVLGGANEGARTAL	100000	20	5000	30000	6	
	GANEGARTALSVVTWNKDIV	100000	20	5000	30000	6	
	SVVTWNKDIVTKITPEGAEE	100000	20	5000	30000	6	
	TKITPEGAEEWSLAIPVMCL	100000	20	5000	30000	6	29946
E3	WSLAIPVMCLLANTTFPCSQ	100000	20	5000	30000	6	
	LANTTFPCSQPPCTPCCYEK	100000	20	5000	30000	6	
	PPCTPCCYEKEPESTLRMLE	100000	20	5000	30000	6	
	EPESTLRMLEDNVMRPGYYQ	100000	20	5000	30000	6	
	DNVMRPGYYQLLKASLTCSP	100000	20	5000	30000	6	
	LLKASLTCSPHRQRRSTKDN	100000	20	5000	30000	6	29964
E2_1	HRQRRSTKDNFNVYKATRPY	100000	20	5000	30000	6	
	FNVYKATRPYLAHCPDCGEG	100000	20	5000	30000	6	
	LAHCPDCGEGHSCHSPVALE	100000	20	5000	30000	6	
	HSCHSPVALERIRNEATDGT	100000	20	5000	30000	6	
	RIRNEATDGTLKIQVSLQIG	100000	20	5000	30000	6	
	LKIQVSLQIGIKTDDSHDWT	100000	20	5000	30000	6	
	IKTDDSHDWTKLRYMDNHMP	100000	20	5000	30000	6	
	KLRYMDNHMPADAERAGLLV	100000	20	5000	30000	6	
	ADAERAGLLVRTSAPCTITG	100000	20	5000	30000	6	
	RTSAPCTITGTMGHFILARC	100000	20	5000	30000	6	29940
E2_2	TMGHFILARCPKGETLTVGF	100000	20	5000	30000	6	
	PKGETLTVGFTDGRKISHSC	100000	20	5000	30000	6	
	TDGRKISHSCTHPFHHDPPV	100000	20	5000	30000	6	
	THPFHHDPPVIGREKFHSRP	100000	20	5000	30000	6	
	IGREKFHSRPQHGKELPCST	100000	20	5000	30000	6	
	QHGKELPCSTYVQSTAATAE	100000	20	5000	30000	6	
	YVQSTAATAEEIEVHMPPDT	100000	20	5000	30000	6	
	EIEVHMPPDTPDRTLMSQQS	100000	20	5000	30000	6	
	PDRTLMSQQSGNVKITVNGQ	100000	20	5000	30000	6	
	GNVKITVNGQTVRYKCNCGG	100000	20	5000	30000	6	29940
E2_3	TVRYKCNCGGSNEGLTTTDK	100000	20	5000	30000	6	
	SNEGLTTTDKVINNCKIDQC	100000	20	5000	30000	6	
	VINNCKIDQCHAAVTNHKKW	100000	20	5000	30000	6	
	HAAVTNHKKWQYNSPLVPRN	100000	20	5000	30000	6	
	QYNSPLVPRNAELGDRKGKI	100000	20	5000	30000	6	
	AELGDRKGKIHIPFPLANVT	100000	20	5000	30000	6	
	HIPFPLANVTCRVPKARNPT	100000	20	5000	30000	6	
	CRVPKARNPTVTYGKNQVIM	100000	20	5000	30000	6	
	VTYGKNQVIMLLYPDHPTLL	100000	20	5000	30000	6	
	LLYPDHPTLLSYRNMGEEPN	100000	20	5000	30000	6	29940
E2_4	SYRNMGEEPNYHEEWVTHKK	100000	20	5000	30000	6	
	YHEEWVTHKKEVRLTVPTEG	100000	20	5000	30000	6	
	EVRLTVPTEGLEVTWGNNEP	100000	20	5000	30000	6	
	LEVTWGNNEPYKYWPQLSTN	100000	20	5000	30000	6	
	YKYWPQLSTNGTAHGHPHEI	100000	20	5000	30000	6	
	GTAHGHPHEIILYYYELYPT	100000	20	5000	30000	6	
	ILYYYELYPTMTVVIVSVAS	100000	20	5000	30000	6	
	MTVVIVSVASFVLLSMVGTA	100000	20	5000	30000	6	
	FVLLSMVGTAVGMCMCARRR	100000	20	5000	30000	6	
	VGMCMCARRRCITPYELTPG	100000	20	5000	30000	6	
	CITPYELTPGATVPFLLSLI	100000	20	5000	30000	6	
	ATVPFLLSLICCIRTAKAAT	100000	20	5000	30000	6	29928
6k	CCIRTAKAATYYEAAAYLWN	100000	20	5000	30000	6	
	YYEAAAYLWNEQQPLFWLQA	100000	20	5000	30000	6	
	EQQPLFWLQALIPLAALIVL	100000	20	5000	30000	6	
	LIPLAALIVLCNCLRLLPCC	100000	20	5000	30000	6	
	CNCLRLLPCCCKTLAFLAVM	100000	20	5000	30000	6	
	CKTLAFLAVMSIGAHTVSAY	100000	20	5000	30000	6	29964
	SIGAHTVSAYEHVTVIPNTV	100000	20	5000	30000	6	
	EHVTVIPNTVGVPYKTLVNR	100000	20	5000	30000	6	
	GVPYKTLVNRPGYSPMVLEM	100000	20	5000	30000	6	

E1_1	PGYSPMVLEMELLSVTLEPT	100000	20	5000	30000	6	
	ELLSVTLEPTLSLDYITCEY	100000	20	5000	30000	6	
	LSLDYITCEYKTVIPSPYVK	100000	20	5000	30000	6	
	KTVIPSPYVKCCGTAECKDK	100000	20	5000	30000	6	
	CCGTAECKDKSLPDYSCKVF	100000	20	5000	30000	6	
	SLPDYSCKVFTGVYPFMWGG	100000	20	5000	30000	6	
	TGVYPFMWGGAYCFCDAENT	100000	20	5000	30000	6	
	AYCFCDAENTQLSEAHVEKS	100000	20	5000	30000	6	29934
E1_2	QLSEAHVEKSESCKTEFASA	100000	20	5000	30000	6	
	ESCKTEFASAYRAHTASASA	100000	20	5000	30000	6	
	YRAHTASASAKLRVLYQGNN	100000	20	5000	30000	6	
	KLRVLYQGNNITVAAYANGD	100000	20	5000	30000	6	
	ITVAAYANGDHAVTVKDAKF	100000	20	5000	30000	6	
	HAVTVKDAKFIVGPMSSAWT	100000	20	5000	30000	6	
	IVGPMSSAWTPFDNKIVVYK	100000	20	5000	30000	6	
	PFDNKIVVYKGDVYNMDYPP	100000	20	5000	30000	6	
	GDVYNMDYPPFGAGRPGQFG	100000	20	5000	30000	6	
	FGAGRPGQFGDIQSRTPESK	100000	20	5000	30000	6	
	DIQSRTPESKDVYANTQLVL	100000	20	5000	30000	6	29934
E1_3	DVYANTQLVLQRPAAGTVHV	100000	20	5000	30000	6	
	QRPAAGTVHVPYSQAPSGFK	100000	20	5000	30000	6	
	PYSQAPSGFKYWLKERGASL	100000	20	5000	30000	6	
	YWLKERGASLQHTAPFGCQI	100000	20	5000	30000	6	
	QHTAPFGCQIATNPVRAVNC	100000	20	5000	30000	6	
	ATNPVRAVNCAVGNIPISID	100000	20	5000	30000	6	
	AVGNIPISIDIPDAAFTRVV	100000	20	5000	30000	6	
	IPDAAFTRVVDAPSLTDMSC	100000	20	5000	30000	6	
	DAPSLTDMSCEVPACTHSSD	100000	20	5000	30000	6	
	EVPACTHSSDFGGVAIIKYA	100000	20	5000	30000	6	
	FGGVAIIKYAASKKGKCAVH	100000	20	5000	30000	6	29934
E1_4	ASKKGKCAVHSMTNAVTIRE	100000	20	5000	30000	6	
	SMTNAVTIREAEIEVEGNSQ	100000	20	5000	30000	6	
	AEIEVEGNSQLQISFSTALA	100000	20	5000	30000	6	
	LQISFSTALASAEFRVQVCS	100000	20	5000	30000	6	
	SAEFRVQVCSTQVHCAAACH	100000	20	5000	30000	6	
	TQVHCAAACHPPKDHIVNYP	100000	20	5000	30000	6	
	PPKDHIVNYPASHTTLGVQD	100000	20	5000	30000	6	
	ASHTTLGVQDISTTAMSWVQ	100000	20	5000	30000	6	
	ISTTAMSWVQKITGGVGLIV	100000	20	5000	30000	6	
	KITGGVGLIVAVAALILIVV	100000	20	5000	30000	6	
	AVAALILIVVLCVSFSRH	100000	20	5000	30000	6	
	IVAVAALILIVVLCVSFSRH	100000	20	5000	30000	6	29928

ICS Pool preparation

Peptide Pool	Peptide Name	Stock Conc. (µg/mL)	Pool Conc. Required (µg/mL)	Dilution Factor	Pool Volume (µL)	Volume of Peptide to Add (µL)	R10 Volume (µL)
Capsid	MEFIPTQTFYNRRYQPRPWT	100000	200	500	800	1.6	
	NRRYQPRPWTPRPTIQVIRP	100000	200	500	800	1.6	
	PRPTIQVIRPRPRPQRQAGQ	100000	200	500	800	1.6	
	RPRPQRQAGQLAQLISAVNK	100000	200	500	800	1.6	
	LAQLISAVNKLTMRAVPQQK	100000	200	500	800	1.6	
	LTMRAVPQQKPRKNRKNKKQ	100000	200	500	800	1.6	
	PRKNRKNKKQKQKQQAPQND	100000	200	500	800	1.6	
	KQKQQAPQNDPKQKKQPPQK	100000	200	500	800	1.6	
	PKQKKQPPQKKPAQKKKKPG	100000	200	500	800	1.6	
	KPAQKKKKPGRRERMCMKIE	100000	200	500	800	1.6	
	RRERMCMKIENDCIFEVKHE	100000	200	500	800	1.6	
	NDCIFEVKHEGKVMGYACLV	100000	200	500	800	1.6	
	GKVMGYACLVGDKVMKPAHV	100000	200	500	800	1.6	
	GDKVMKPAHVKGTIDNADLA	100000	200	500	800	1.6	
	KGTIDNADLAKLAFKRSSKY	100000	200	500	800	1.6	
	KLAFKRSSKYDLECAQIPVH	100000	200	500	800	1.6	
	DLECAQIPVHMKSDASKFTH	100000	200	500	800	1.6	
	MKSDASKFTHEKPEGYYNWH	100000	200	500	800	1.6	
	EKPEGYYNWHHGAVQYSGGR	100000	200	500	800	1.6	
	HGAVQYSGGRFTIPTGAGKP	100000	200	500	800	1.6	
	FTIPTGAGKPGDSGRPIFDN	100000	200	500	800	1.6	
	GDSGRPIFDNKGRVVAIVLG	100000	200	500	800	1.6	
	KGRVVAIVLGGANEGARTAL	100000	200	500	800	1.6	
	GANEGARTALSVVTWNKDIV	100000	200	500	800	1.6	
	SVVTWNKDIVTKITPEGAEE	100000	200	500	800	1.6	
	TKITPEGAEEWSLAIPVMCL	100000	200	500	800	1.6	758.4
E3	WSLAIPVMCLLANTTFPCSQ	100000	200	500	800	1.6	
	LANTTFPCSQPPCTPCCYEK	100000	200	500	800	1.6	
	PPCTPCCYEKEPESTLRMLE	100000	200	500	800	1.6	
	EPESTLRMLEDNVMRPGYYQ	100000	200	500	800	1.6	
	DNVMRPGYYQLLKASLTCSP	100000	200	500	800	1.6	
	LLKASLTCSPHRQRRSTKDN	100000	200	500	800	1.6	790.4
	HRQRRSTKDNFNVYKATRPY	100000	200	500	800	1.6	
	FNVYKATRPYLAHCPDCGEG	100000	200	500	800	1.6	
	LAHCPDCGEGHSCHSPVALE	100000	200	500	800	1.6	
	HSCHSPVALERIRNEATDGT	100000	200	500	800	1.6	
	RIRNEATDGTLKIQVSLQIG	100000	200	500	800	1.6	
	LKIQVSLQIGIKTDDSHDWT	100000	200	500	800	1.6	
	IKTDDSHDWTKLRYMDNHMP	100000	200	500	800	1.6	
	KLRYMDNHMPADAERAGLLV	100000	200	500	800	1.6	
	ADAERAGLLVRTSAPCTITG	100000	200	500	800	1.6	

	RTSAPCTITGTMGHFILARC	100000	200	500	800	1.6	
	TMGHFILARCPKGETLTVGF	100000	200	500	800	1.6	
	PKGETLTVGFTDGRKISHSC	100000	200	500	800	1.6	
	TDGRKISHSCTHPFHHDPPV	100000	200	500	800	1.6	
	THPFHHDPPVIGREKFHSRP	100000	200	500	800	1.6	
	IGREKFHSRPQHGKELPCST	100000	200	500	800	1.6	
	QHGKELPCSTYVQSTAATAE	100000	200	500	800	1.6	
	YVQSTAATAEEIEVHMPPDT	100000	200	500	800	1.6	
	EIEVHMPPDTPDRTLMSQQS	100000	200	500	800	1.6	
	PDRTLMSQQSGNVKITVNGQ	100000	200	500	800	1.6	
	GNVKITVNGQTVRYKCNCGG	100000	200	500	800	1.6	
E2	TVRYKCNCGGSNEGLTTTDK	100000	200	500	800	1.6	
	SNEGLTTTDKVINNCKIDQC	100000	200	500	800	1.6	
	VINNCKIDQCHAAVTNHKKW	100000	200	500	800	1.6	
	HAAVTNHKKWQYNSPLVPRN	100000	200	500	800	1.6	
	QYNSPLVPRNAELGDRKGKI	100000	200	500	800	1.6	
	AELGDRKGKIHIPFPLANVT	100000	200	500	800	1.6	
	HIPFPLANVTCRVPKARNPT	100000	200	500	800	1.6	
	CRVPKARNPTVTYGKNQVIM	100000	200	500	800	1.6	
	VTYGKNQVIMLLYPDHPTLL	100000	200	500	800	1.6	
	LLYPDHPTLLSYRNMGEEPN	100000	200	500	800	1.6	
	SYRNMGEEPNYHEEWVTHKK	100000	200	500	800	1.6	
	YHEEWVTHKKEVRLTVPTEG	100000	200	500	800	1.6	
	EVRLTVPTEGLEVTWGNNEP	100000	200	500	800	1.6	
	LEVTWGNNEPYKYWPQLSTN	100000	200	500	800	1.6	
	YKYWPQLSTNGTAHGHPHEI	100000	200	500	800	1.6	
	GTAHGHPHEIILYYYELYPT	100000	200	500	800	1.6	
	ILYYYELYPTMTVVIVSVAS	100000	200	500	800	1.6	
	MTVVIVSVASFVLLSMVGTA	100000	200	500	800	1.6	
	FVLLSMVGTAVGMCMCARRR	100000	200	500	800	1.6	
	VGMCMCARRRCITPYELTPG	100000	200	500	800	1.6	
	CITPYELTPGATVPFLLSLI	100000	200	500	800	1.6	
	ATVPFLLSLICCIRTAKAAT	100000	200	500	800	1.6	732.8
	CCIRTAKAATYYEAAAYLWN	100000	200	500	800	1.6	
	YYEAAAYLWNEQQPLFWLQA	100000	200	500	800	1.6	
6k	EQQPLFWLQALIPLAALIVL	100000	200	500	800	1.6	
	LIPLAALIVLCNCLRLLPCC	100000	200	500	800	1.6	
	CNCLRLLPCCCKTLAFLAVM	100000	200	500	800	1.6	
	CKTLAFLAVMSIGAHTVSAY	100000	200	500	800	1.6	790.4
	SIGAHTVSAYEHVTVIPNTV	100000	200	500	800	1.6	
	EHVTVIPNTVGVPYKTLVNR	100000	200	500	800	1.6	
	GVPYKTLVNRPGYSPMVLEM	100000	200	500	800	1.6	
	PGYSPMVLEMELLSVTLEPT	100000	200	500	800	1.6	
	ELLSVTLEPTLSLDYITCEY	100000	200	500	800	1.6	
	LSLDYITCEYKTVIPSPYVK	100000	200	500	800	1.6	
	KTVIPSPYVKCCGTAECKDK	100000	200	500	800	1.6	
	CCGTAECKDKSLPDYSCKVF	100000	200	500	800	1.6	
	SLPDYSCKVFTGVYPFMWGG	100000	200	500	800	1.6	
	TGVYPFMWGGAYCFCDAENT	100000	200	500	800	1.6	
	AYCFCDAENTQLSEAHVEKS	100000	200	500	800	1.6	
	QLSEAHVEKSESCKTEFASA	100000	200	500	800	1.6	
	ESCKTEFASAYRAHTASASA	100000	200	500	800	1.6	
	YRAHTASASAKLRVLYQGNN	100000	200	500	800	1.6	
	KLRVLYQGNNITVAAYANGD	100000	200	500	800	1.6	
	ITVAAYANGDHAVTVKDAKF	100000	200	500	800	1.6	
	HAVTVKDAKFIVGPMSSAWT	100000	200	500	800	1.6	
	IVGPMSSAWTPFDNKIVVYK	100000	200	500	800	1.6	
	PFDNKIVVYKGDVYNMDYPP	100000	200	500	800	1.6	
	GDVYNMDYPPFGAGRPGQFG	100000	200	500	800	1.6	
	FGAGRPGQFGDIQSRTPESK	100000	200	500	800	1.6	
	DIQSRTPESKDVYANTQLVL	100000	200	500	800	1.6	
E1	DVYANTQLVLQRPAAGTVHV	100000	200	500	800	1.6	
	QRPAAGTVHVPYSQAPSGFK	100000	200	500	800	1.6	
	PYSQAPSGFKYWLKERGASL	100000	200	500	800	1.6	
	YWLKERGASLQHTAPFGCQI	100000	200	500	800	1.6	
	QHTAPFGCQIATNPVRAVNC	100000	200	500	800	1.6	
	ATNPVRAVNCAVGNIPISID	100000	200	500	800	1.6	
	AVGNIPISIDIPDAAFTRVV	100000	200	500	800	1.6	
	IPDAAFTRVVDAPSLTDMSC	100000	200	500	800	1.6	
	DAPSLTDMSCEVPACTHSSD	100000	200	500	800	1.6	
	EVPACTHSSDFGGVAIIKYA	100000	200	500	800	1.6	
	FGGVAIIKYAASKKGKCAVH	100000	200	500	800	1.6	
	ASKKGKCAVHSMTNAVTIRE	100000	200	500	800	1.6	
	SMTNAVTIREAEIEVEGNSQ	100000	200	500	800	1.6	
	AEIEVEGNSQLQISFSTALA	100000	200	500	800	1.6	
	LQISFSTALASAEFRVQVCS	100000	200	500	800	1.6	
	SAEFRVQVCSTQVHCAAACH	100000	200	500	800	1.6	
	TQVHCAAACHPPKDHIVNYP	100000	200	500	800	1.6	
	PPKDHIVNYPASHTTLGVQD	100000	200	500	800	1.6	
	ASHTTLGVQDISTTAMSWVQ	100000	200	500	800	1.6	
	ISTTAMSWVQKITGGVGLIV	100000	200	500	800	1.6	
	KITGGVGLIVAVAALILIVV	100000	200	500	800	1.6	
	AVAALILIVVLCVSFSRH	100000	200	500	800	1.6	
	IVAVAALILIVVLCVSFSRH	100000	200	500	800	1.6	728

Figure S1. ICS gating Strategy

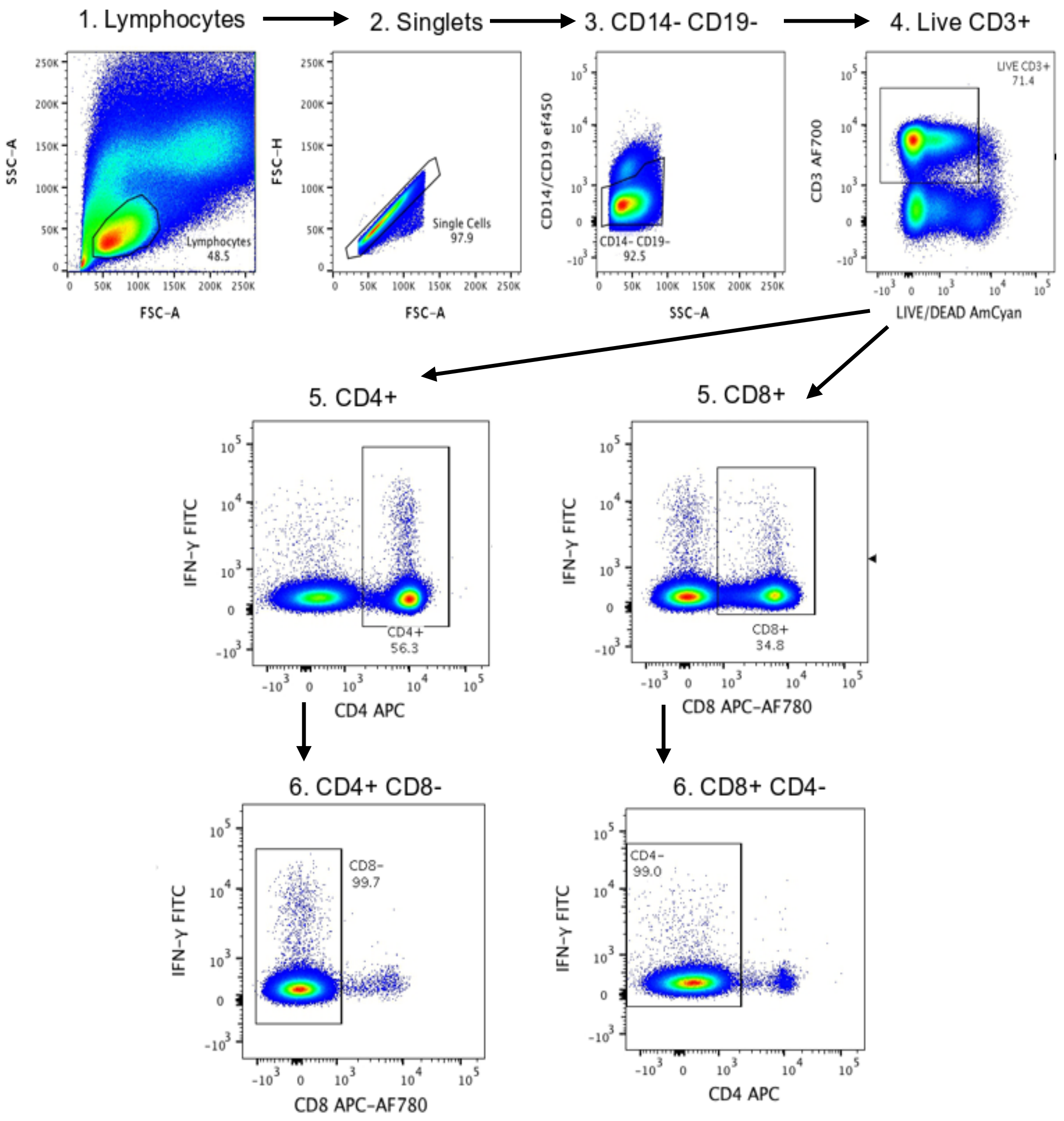

Figure S2. Maximum PRNT50 neutralisation titres against four CHIKV lineages

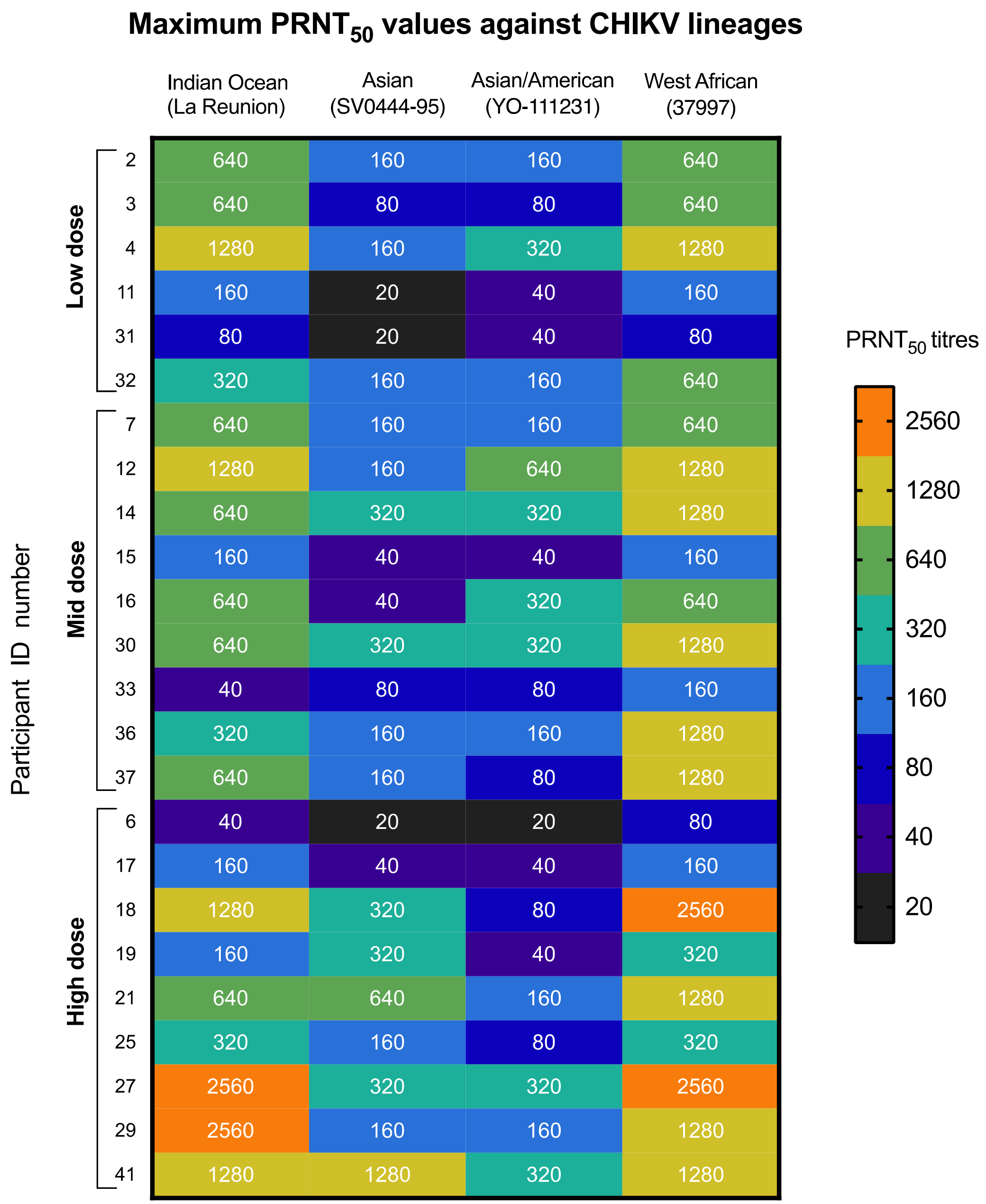

CONFIDENTIAL

UNIVERSITY OF OXFORD

A phase I study to determine the safety and immunogenicity of the candidate Chikungunya Virus (CHIKV) vaccine ChAdOx1 Chik in healthy adult volunteers

Study Reference: CHIK001

Protocol Number: v2.0

Date: 17th July 2018

Chief Investigator: Professor A.V.S Hill

Sponsor: University of Oxford

Funder: Innovate UK

REC Number: 18/SC/0004

EudraCT Number: 2017-004483-35

IRAS Reference: 235713

©Oxford University

PAGE LEFT INTENTIONALLY BLANK

©Oxford University

**Full Study Title**	A phase I clinical trial to determine the safety and immunogenicity of the candidate CHIKV vaccine ChAdOx1 Chik in UK healthy adult volunteers.<br>**Study Code**: CHIK001<br>**EudraCT Number**: 2017-004483-35<br>**IRAS project ID**: 235713
**Chief Investigator**	**Prof Adrian V.S. Hill**<br>Centre for Clinical Vaccinology and Tropical Medicine<br>University of Oxford,<br>Churchill Hospital, Old Road, Headington<br>Oxford, OX3 7LE<br>Email: adrian.hill@ndm.ox.ac.uk
**Trial Sites**	**Centre for Clinical Vaccinology and Tropical Medicine**<br>Churchill Hospital, Old Road, Headington<br>Oxford, OX3 7LE
**Sponsoring Institution**	**University of Oxford**<br>Clinical Trials and Research Governance<br>Joint Research Office, Block 60, Churchill Hospital,<br>Old Road, Headington, Oxford, OX3 7LE<br>Tel: 01865 572224<br>Fax: 01865 572228<br>Email: ctrg@admin.ox.ac.uk
**External Monitor**	**Clinical Trials & Research Governance**<br>Joint Research Office, Block 60,<br>Churchill Hospital,<br>Old Road, Headington, Oxford OX3 7LE
**Local Safety Monitor (Chairman of Local Safety Committee)**	**Dr Brian Angus**<br>Centre for Clinical Vaccinology and Tropical Medicine<br>Churchill Hospital, Old Road, Headington<br>Oxford, OX3 7LE<br>Tel: 01865 220289<br>Email: brian.angus@ndm.ox.ac.uk

©Oxford University

Confidentiality Statement

This document contains confidential information that must not be disclosed to anyone other than the Sponsor, the Investigator Team, HRA, host organisation, members of the Research Ethics Committee and other regulatory bodies. This information cannot be used for any purpose other than the evaluation or conduct of the clinical investigation without the prior written consent of Professor Adrian Hill.

Statement of Compliance

The trial will be conducted in compliance with the protocol, the principles Good Clinical Practice Guideline , Medicines for Human Use (Clinical Trial) Regulations 2004 (as amended) and all other applicable regulatory requirements.

Chief Investigator Approval and Agreement

I have read the trial protocol and agree to conduct the trial in compliance with the protocol, the principles of Good Clinical Practice and all applicable regulatory requirements.

I hereby approve this version of the protocol and declare no conflict of interest

Adrian Hill

-- -- --------------------------------

Chief Investigator

Name **Signature** **Date**

©Oxford University

Modification History

Version	Date	Author(s)	Modifications
1.0	05.11.2017	Pedro Folegatti, Mark Tilley, Cesar Lopez-Camacho, Arturo Reyes-Sandoval, Adrian Hill	N/A
2.0	17.07.2018		To increase the volunteer payments for travel from £10 to £15

©Oxford University

Table of Contents

1. SYNOPSIS 9

2. ABBREVIATIONS 11

3. INTRODUCTION 13

3.1 Background 13

3.2 The need for and progress towards a CHIKV vaccine 14

3.3 The Chik insert 14

3.4 Adenovirus-vectored Vaccines 15

3.5 ChAdOx1 16

3.6 Development of ChAdOx1 Chik 17

3.7 Preclinical Studies 17

3.7.1 Efficacy and Immunogenicity 17

3.8 Previous clinical experience 19

3.8.1 Clinical Usage of CHIKV Proteins in Vaccine Trials 19

3.8.2 Clinical Usage of the ChAdOx1 Vector in Vaccine Trials 23

3.9 Rationale 24

3.10 Vaccine Development Strategy 25

4. OBJECTIVES AND ENDPOINTS 26

4.1 Primary Objective 26

4.1.1 Primary Outcome Measures 26

4.2 Secondary Objective 26

4.2.1 Secondary Outcome Measures 26

5. STUDY OVERVIEW 27

5.1 Rationale for Selected Doses 27

5.2 Study Groups 28

5.2.1 First Volunteers 28

5.2.2 Duration of study 28

5.2.3 Definition of Start and End of Trial 28

5.3 Potential Risks for volunteers 29

5.4 Known Potential Benefits 29

6. RECRUITMENT AND WITHDRAWAL OF TRIAL VOLUNTEERS 30

6.1 Volunteers 30

6.2 Informed consent 30

6.3 Inclusion and exclusion criteria 31

6.3.1 Inclusion Criteria 31

6.3.2 Exclusion Criteria 31

6.3.3 Effective contraception for female volunteers 32

6.3.4 Prevention of 'Over Volunteering' 33

6.3.5 Criteria for postponement of vaccination 33

©Oxford University

6.3.6 Withdrawal of Volunteers 33
6.4 Compliance with Dosing Regime 34
6.5 Pregnancy 34
7. CLINICAL PROCEDURES 35
7.1 Study procedures 35
7.2 Observations 35
7.3 Blood Tests and Urinalysis 35
7.4 Study visits 36
7.4.1 Screening visit 36
7.4.2 Day 0: Enrolment and Vaccination Visit 37
7.4.2.1 Vaccinations 37
7.4.2.2 Sequence of Enrolment and Vaccination of Volunteers 38
7.4.3 Subsequent visits: days 2, 7, 14, 28, 56 and 182. 39
8. INVESTIGATIONAL PRODUCTS 41
8.1. Manufacturing and Presentation 41
8.1.1 Description of ChAdOx1 Chik 41
8.1.2. ChAdOx1 Chik formulation and packaging 41
8.2 Supply 41
8.3 Storage 41
8.4 Administration of Investigational Medicinal Products 41
8.5 Minimising environmental contamination with genetically modified organisms (GMO) 42
9. ASSESSMENT OF SAFETY 43
9.1 Definitions 43
9.1.1 Adverse Event (AE) 43
9.1.2 Adverse Reaction (AR) 43
9.1.3 Unexpected Adverse Reaction 43
9.1.4 Serious Adverse Event (SAE) 43
9.1.5 Serious Adverse Reaction (SAR) 44
9.1.6 Suspected Unexpected Serious Adverse Reaction (SUSAR) 44
9.2 Foreseeable Adverse Reactions: 44
9.3 Expected Serious Adverse Events 44
9.4 Causality Assessment 44
9.5 Reporting Procedures for All Adverse Events (see SOP VC027) 45
9.5.1 Reporting Procedures for Serious AEs (see SOP OVC005 Safety Reporting) 45
9.5.2 Reporting Procedures for SUSARS 46
9.5.3 Development Safety Update Report 46
9.6 Assessment of severity 46
9.7 Procedures to be followed in the event of abnormal findings 48
9.8 Local Safety Committee 48

©Oxford University

9.8.1 Interim Safety Reviews 49
9.9 Safety Stopping/Holding Rules 49
9.9.1 Group holding rules 49
10. STATISTICS 51
11. DATA MANAGEMENT 52
11.1 Data Handling 52
11.2 Record Keeping 52
11.3 Source Data and Case Report Forms (CRFs) 52
11.4 Data Protection 52
11.5 Data Quality 53
12. QUALITY CONTROL AND QUALITY ASSURANCE PROCEDURES 54
12.1 Investigator procedures 54
12.2 Monitoring 54
12.3 Protocol deviation 54
12.4 Audit & inspection 54
13. SERIOUS BREACHES 55
14. ETHICS AND REGULATORY CONSIDERATIONS 56
14.1 Declaration of Helsinki 56
14.2 Guidelines for Good Clinical Practice 56
14.3 Approvals 56
14.4 Volunteer Confidentiality 56
15. FINANCING AND INSURANCE 58
15.1 Financing 58
15.2 Insurance 58
15.3 Compensation 58
16. PUBLICATION POLICY 59
17. REFERENCES 60

©Oxford University

1. SYNOPSIS

**Trial Title**	A phase I study to determine the safety and immunogenicity of the candidate Chikungunya Virus (CHIKV) vaccine ChAdOx1 Chik in healthy adult volunteers.
**Trial Centre**	Centre for Clinical Vaccinology & Tropical Medicine, University of Oxford, Churchill Hospital, Old Road, Headington, Oxford, OX3 7LE
**Trial Identifier**	CHIK001
**Clinical phase**	I
**Study Design**	Open–labelled, non-randomised, dose escalation, first-in-human, single centre, phase I clinical trial
**Population**	Healthy adults aged 18 – 50 years
**Planned Sample Size**	24 volunteers

Group	Dose of ChAdOx1 Chik
Group 1 (n=6)	5×10^{9} vp
Group 2 (n=9)	2.5×10^{10} vp
Group 3 (n=9)	5×10^{10} vp

**Follow-up duration**	26 weeks post vaccine administration
**Planned Trial Period**	Q1 2018 to Q1 2019
**Primary Objective**	To assess the safety profile of the candidate vaccine ChAdOx1 Chik in healthy adult volunteers
**Secondary Objective**	To assess the immunogenicity of the candidate vaccine ChAdOx1 Chik in healthy adult volunteers

©Oxford University

**Investigational Products**	ChAdOx1 Chik, a replication-deficient simian adenoviral vector expressing whole structural gene cassette for the precursors of viral structural proteins (C-E3-E2-6K-E1) of the CHIKV.
**Dose per Administration**	ChAdOx1 Chik 5 x 10^9 vp<br>ChAdOx1 Chik 2.5 x 10^{10} vp<br>ChAdOx1 Chik 5 x 10^{10} vp
**Form**	Liquid (all finished products)
**Route**	Intramuscularly (IM) into the deltoid region of the arm

©Oxford University

2. ABBREVIATIONS

AE	Adverse event
AR	Adverse reaction
CBF	Clinical Biomanufacturing Facility
CCVTM	Centre for Clinical Vaccinology and Tropical Medicine
ChAdOx1	Chimpanzee Adenovirus Ox1
ChAdOx1 Chik	Recombinant Chimpanzee Adenovirus Ox1 with CHIKV structural proteins
CHIKV	Chikungunya virus
CI	Chief Investigator
CRF	Case Report Form or Clinical Research Facility
CTRG	Clinical Trials Research Governance
DSUR	Development Safety Update Report
ELISA	Enzyme linked immunosorbent assay
ELISpot	Enzyme linked immunospot assay
FBC	Full blood count
GCP	Good Clinical Practice
GMO	Genetically modified organism
GMP	Good Manufacturing Practice
HBsAg	Hepatitis B surface antigen
HCG	Human Chorionic Gonadotrophin
HCV	Hepatitis C virus
HIV	Human Immunodeficiency virus
HLA	Human leukocyte antigen
IB	Investigators Brochure
ICH	International Conference on Harmonisation
IM	Intramuscular
IMP	Investigational Medicinal Product
LSC	Local Safety Committee
LSM	Local Safety Monitor
MHRA	Medicines and Healthcare products Regulatory Agency
MVA	Modified Vaccinia Virus Ankara
pfu	plaque forming units
PIS	Participant information sheet
PBMC	Peripheral blood mononuclear cells
QP	Qualified Person

©Oxford University

REC	Research Ethics Committee
SAE	Serious Adverse Event
SAR	Serious Adverse Reaction
SFU	Spot forming units
SOP	Standard Operating Procedure
SUSAR	Suspected Unexpected Serious Adverse Reaction
TMF	Trial Master File
vp	Viral particles
WHO	World Health Organisation

©Oxford University

3. INTRODUCTION

3.1 Background

Chikungunya fever is an arbovirus disease caused by the Chikungunya virus (CHIKV), a mosquito-borne alphavirus. Alphaviruses are a genus of enveloped, positive sense, single-stranded RNA viruses, belonging to the *Togaviridae* family. The disease typically consists of an acute illness characterised by fever, rash, myalgia and generally symmetrical and peripheral, often incapacitating, polyarthralgia and/or polyarthritis, which usually lasts weeks to months or years (1). Fatal disease is rare, but has been observed in neonates and elderly individuals, as well as in those with comorbities such as diabetes or cardiovascular, respiratory, and neurologic disorders.

CHIKV has been identified in over 100 countries in Asia, Africa, Oceania, Europe and the Americas (2, 3). The virus was first isolated during an outbreak on the Makonde Plateau in Tanzania in 1952–53. Outbreaks were subsequently identified in Asia during the 1950s and 1960s. During the past 50 years, numerous CHIKV re-emergences have been documented in both Africa and Asia, with irregular intervals between outbreaks. In 2004, CHIKV emerged in Kenya and spread to Comoros, where 5,000 cases were reported. In 2005–2006, the outbreak spread to other islands in the Indian Ocean with an estimated 300,000 cases of CHIKV infections and 237 resultant deaths (4) and an estimated economic cost of €43.9 million (5). Since 2005, India, Indonesia, Maldives, Myanmar and Thailand have reported over 1.9 million cases and in 2007, the virus reached Europe and caused the first autochthonous epidemic outbreak in the north-east of Italy. CHIKV has since spread to the Caribbean with the first case reported in Saint Martin in 2013 with local transmission now confirmed in over 43 countries and territories in the World Health Organization (WHO) Region of the Americas. In 2015, over 1 million suspected cases of Chikungunya have been recorded in the Caribbean islands, Latin American countries, and the United States of America with 191 deaths during the same period. Canada, Mexico and USA have also recorded imported cases (3, 6-8).

Transmission occurs through the bite of infected female *Aedes* mosquitos which are widely distributed across the globe. *A. aegypti* are found in the tropics and subtropics whereas *A. albopictus* have a wider distribution (also being found in temperate regions) and has spread from Asia to become established in areas of Africa, Europe and the Americas, causing the spread of CHIKV to new geographical regions (3, 6).

CHIKV is listed as priority pathogen by the UK Vaccine Network and the US National Institute of Allergy and Infectious Diseases (NIAID - category C). It has been designated as a serious condition requiring action by the WHO to promote Research and Development to control future outbreaks. There are currently no available treatments or licensed vaccines.

©Oxford University

3.2 The need for and progress towards a CHIKV vaccine

Vector control measures are frequently complex and have proven to be insufficient in the containment of arbovirus infections outbreaks. Effective mosquito control strategies often rely on adequate staffing levels (entomologists, social scientists, operational vector-control staff), technical expertise at decentralised levels of services, funding availability, adequate geographical coverage, insecticide use (and emerging resistance patterns), community engagement, capacity building and monitoring & evaluation. These requirements are frequently unmet, even in developed countries (9).

There are no licensed drugs to curb CHIKV replication and improve clinical outcome, and only standard symptomatic treatment is available. Chronic arthralgia can lead to persistent incapacitation, requiring long-term treatment with nonsteroidal anti-inflammatory and immunosuppressive drugs. Several classes of direct-acting and host-targeting antivirals have been reported to have *in vitro* activity against CHIKV. However, *in vivo* efficacy has not yet been evaluated in animal models for most of these drugs. Chloroquine is an antimalarial drug, known to have *in vitro* antiviral activity against the virus. However, clinical trials of chloroquine in CHIKV-infected patients could not prove its efficacy for treatment of CHIKV infection (10, 11).

Humans are thought to be the only amplification hosts during urban transmission. Therefore, the most cost-effective means of controlling the spread of the infection is by vaccination (12). There is currently no licensed vaccine against CHIKV and there are several vaccine candidates in pre-clinical development but only a few have entered clinical trials. The first live-attenuated vaccine, known as 181/clone25 or TSIGSD-218, progressed the furthest into clinical trials. The vaccine was highly immunogenic but produced arthralgia in some vaccines and its further development was discontinued because of safety concerns after reversal of the attenuation had been reported both in mice and humans. Virus-like particle (VLP) and live-vectored vaccines have successfully elicited immune responses in phase I clinical trials and are now entering phase II studies (13). However, VLP vaccines usually require support of adjuvants and multiple injections to induce seroconversion and reach high antibody titres, increasing the costs and limiting their use in low-income countries. The use of replication competent recombinant live attenuated virus carry the risks of inadequate attenuation causing disseminated disease, particularly in immunocompromised hosts. Replication deficient vectors, however, avoid that risk while maintaining the advantages of native antigen presentation, elicitation of T cell immunity and the ability to express multiple antigens (14).

3.3 The Chik insert

CHIKV belongs to genus Alphavirus (family Togaviridae) and has a positive-strand RNA genome approximately 12 kb in length. A large 5′ open reading frame (ORF) encodes for a non-structural (ns) polyprotein designated as P1234. The second ORF encodes for the

©Oxford University

precursors of viral structural proteins known as Capsid (C), Envelope (E) E3, E2, the 6K protein and E1 (Figure 1)

Since our objective is to induce neutralising antibodies we have focused at producing a ChAdOx1 Chik vaccine carrying the whole structural gene cassette (C-E3-E2-6K-E1), from which immunogenicity is derived. This cassette is well characterised and in other vectors, has elicited a CHIKV specific immunogenic response in several pre-clinical and clinical vaccine developments and in several vaccine platforms in which it has been demonstrated to induce good levels of immunogenicity in other early phase human vaccine trials (15, 16).

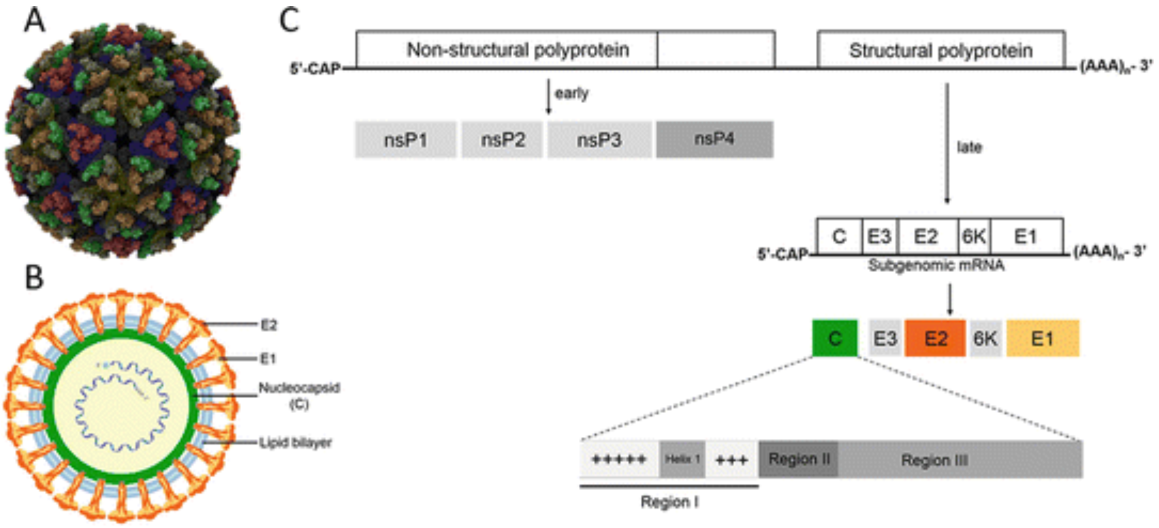

Figure 1: Genomic structure of Chikungunya Virus. Image taken from: Metz S.W., Pijlman G.P. (2016) Function of Chikungunya Virus Structural Proteins. In: Okeoma C. (eds) Chikungunya (17).

3.4 Adenovirus-vectored Vaccines

Adenoviruses are attractive vectors for human vaccination. They possess a stable genome so that inserts of foreign genes are not deleted and they can infect large numbers of cells without any evidence of insertional mutagenesis.

Replication defective adenovirus can be engineered by deletion of genes from the E1 locus, which is required for viral replication, and these viruses can be propagated easily with good yields in cell lines expressing E1 from AdHu5 such as human embryonic kidney cells 293 (HEK 293 cells) (18).

Previous mass vaccination campaigns in over 2 million adult US military personnel using orally administered live human adenovirus serotype 4 and 7 have shown good safety and efficacy data (19). Human adenoviruses are under development as vectors for malaria, HIV and hepatitis C vaccines, amongst others. They have been used extensively in human trials with excellent safety profile mainly as vectors for HIV vaccines.

A limiting factor to widespread use of human adenovirus as vaccine vectors has been the level of anti-vector immunity present in humans where adenovirus is a ubiquitous infection.

©Oxford University

The prevalence of immunity to human adenoviruses prompted the consideration of simian adenoviruses as vectors, as they exhibit hexon structures homologous to human adenoviruses (20). Simian adenoviruses are not known to cause pathological illness in humans and the prevalence of antibodies to chimpanzee origin adenoviruses is less than 5% in humans residing in the US.

In chimpanzee adenoviruses, the E1 locus can be deleted to render viruses replication deficient and allow transcomplementation on an E1 AdHu5 complementing cell line (21). Whilst they exhibit hexon structures homologous to that of human adenoviruses (22), the lack of sequence homology at the E1 flanking sequence prevents homologous recombination and production of replication competent virus (23)

Chimpanzee adenoviral vectors can be manufactured cost-effectively and are now in clinical development as possible vaccines against malaria, HIV, tuberculosis, influenza, hepatitis C, RSV, Cancer and Ebola.

3.5 ChAdOx1

ChAdOx1 is a novel recombinant chimpanzee adenovirus designed as a vaccine vector, developed by The Jenner Institute at the University of Oxford. This viral vector has been used by researchers at the University of Oxford to produce a number of vaccines expressing a range of different antigens. Three phase I clinical trials have been completed in the UK using ChAdOx1 with different inserts (two influenza trials and one TB trial) and there are two ongoing clinical trials using malaria and prostate cancer inserts.

ChAdOx1 is produced from a replication-deficient (E1 and E3 deleted) simian adenovirus and it has been described by Dicks et al (24). The vector was constructed in a bacterial artificial chromosome (BAC) to facilitate genetic manipulation of genomic clones with improved stability and flexibility. Cellular immunogenicity of recombinant E1 E3-deleted ChAdOx1 was comparable to that of other species E derived chimpanzee adenovirus vectors including ChAd63, the first simian adenovirus vector to enter clinical trials in humans. The E1 region is essential for viral replication, hence the ability to delete E1 renders the new vector immediately replication incompetent. The deletion of the non-essential adenovirus E3 region increases the insert capacity of the new vector by approximately 5kb. It is known that the proteins encoded by the E4 region of adenoviruses interact with E1 during viral replication, and the imperfect interaction between the gene products of the AdHu5 E1 gene produced by HEK293 cells and simian E4 gene products has been found to result in impaired viral replication in this cell line, and consequently lower virus yields. In ChAdOx1, Ad5 E4Orf4 has been inserted to replace the homologous simian virus coding sequence, resulting in improved viral replication during vaccine production. Since no replication of the virus takes place after immunization, this replacement has no effect on immunogenicity of the viral vector. Insertion of recombinant antigens at the E1 locus is performed using Gateway® site specific recombination technology (Invitrogen).

©Oxford University

3.6 Development of ChAdOx1 Chik

ChAdOx1 Chik comprises of the ChAdOx1 vector cloned into which is the full structural genome of CHIKV, being Capsid (C), Envelope (E3, E2 and E1) and the 6K protein. This cassette was prepared by Gateway® recombination of an entry plasmid encoding the C-E3-E2-6K-E1 CHIKV structural proteins and the E1-and E3-deleted ChAdOx1 destination vector.

3.7 Preclinical Studies

3.7.1 Efficacy and Immunogenicity

Balb/c mice were prime vaccinated with ChAdOX1 Chik or an unrelated control and serum samples then taken after 14 days, with endpoint titres measured by ELISA and ELISPOT. High levels of anti-CHIKV antibodies were measured by ELISA (**Error! Reference source not found.**), as well as high levels of cellular responses by ELISPOT (**Figure 2**: B-cell Immunogenicity of ChAdOx1 Chik (anti-CHIKV antibodies). The reciprocal titer was calculated using the mean of the OD values of the control sera plus 3 times the standard deviation of that mean.) at day 14 after vaccination (Cesar Lopez-Camacho, unpublished data).

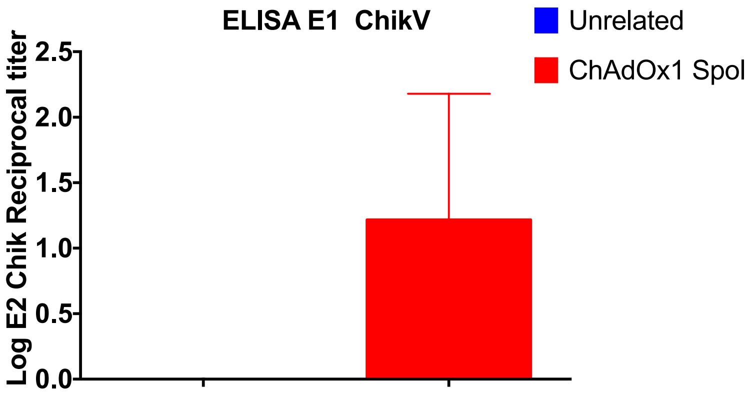

Figure 2: B-cell Immunogenicity of ChAdOx1 Chik (anti-CHIKV antibodies). The reciprocal titer was calculated using the mean of the OD values of the control sera plus 3 times the standard deviation of that mean.

Functional CHIKV neutralisation assays have assessed sera taken from vaccinated mice at day 14 post immunisation and used in a model of CHIKV infection *in vitro*. These assays show that ChAdOx1 Chik is capable of generating Neutralisation titers (NT50) against CHIKV (**Figure 2**) and furthermore, these NT50 titers are greater than those achieved in comparable assays using another vaccine currently in Phase II clinical trial (NCT03101111) that uses multiple immunisations (25) (Cesar Lopez-Camacho, unpublished data).

©Oxford University

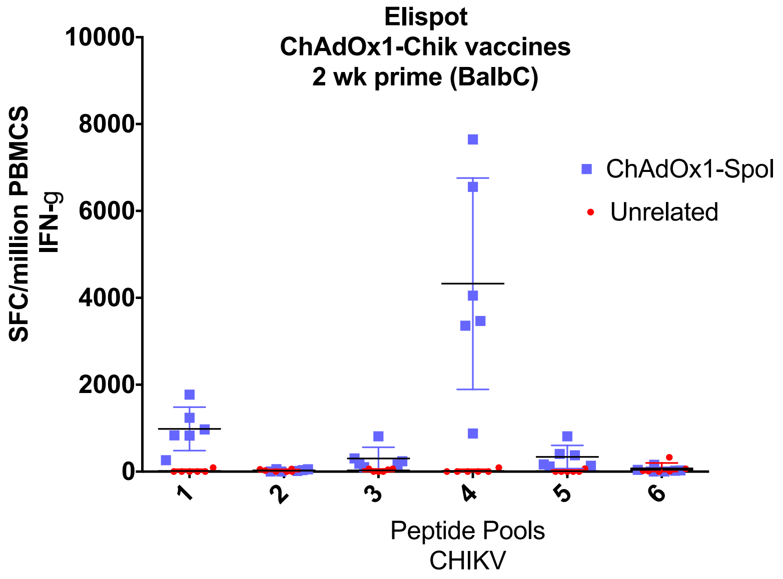

Figure 3: T-Cell Immunogenicity of ChAdOx1 Chik measured by ELISPOT assay. Results are expressed as the Spot forming cells (SFC) per million PBMCs.

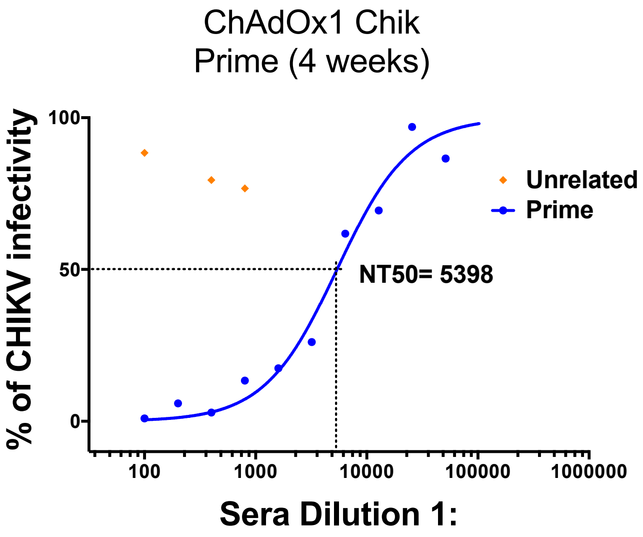

	Prime
Best-fit values	
LogEC50	3.732
HillSlope	1.332
EC50	5398
Std. Error	
LogEC50	0.05702
HillSlope	0.2061
95% Confidence Intervals	
LogEC50	3.603 to 3.861
HillSlope	0.8663 to 1.799
EC50	4011 to 7265
Goodness of Fit	
Degrees of Freedom	9
R square	0.9707
Absolute Sum of Squares	496.8
Sy.x	7.430

Figure 2. CHIKV neutralisation assay. Different dilutions of ChAdOx1 Chik mice sera and unrelated control were mixed with CHIKV and incubated for 1h. Later the mix was added to cell cultures that are permissive to CHIKV infection. Plaque numbers were counted and % of infectivity was calculated based on the percentage of plaque formation from Media with FCS only. NT50 was calculated after a non-linear regression fit.

©Oxford University

3.8 Previous clinical experience

3.8.1 Clinical Usage of CHIKV Proteins in Vaccine Trials

This is a First-in-Human clinical trial of the ChAdOx1 Chik vaccine, which carries CHIKV proteins C-E3-E2-6K-E1. These CHIKV structural proteins have been employed in seven other vaccine clinical trials where they have elicited an immune response with no reported serious adverse events attributed to the structural proteins as described in the below The ChAdOx1 Chik vaccine most probably forms a structure similar to those of MV-CHIK-022 and VRC-CHKVLP059-00-VP, since it too is comprised solely of structural proteins. Being non-replicative, the vaccine design aims to avoid the AEs of the live-attenuated vaccine.

©Oxford University

Table 1: Clinical Experience with CHIKV structural proteins

CHIKV vaccine construct	Sponsor	Phase	Trial ID	Regimen[a]	Dose	n.	AEs	Reference
Live recombinant measles-virus-based CHIKV vaccine carrying CHIKV C-E3-E2-6K-E1 proteins. (MV-CHIK-022)	Thermis GmbH / Institut Pasteur	1	NCT03028441	P-B	1·5 × 104 TCID50 per 0·05 mL	12	0	Lancet Infect Dis. 2015 May;15(5):519-27.
				P-B	7·5 × 104 TCID50 per 0·25 mL	12	0	
				P-B	3·0 × 105 TCID50 per 1·0 mL	12	0	
Lentiviral Virus-Like-Particle carrying CHIKV E3-E2-6K-E1 proteins. (VRC-CHKVLP059-00-VP)	NIAID	1	NCT01489358	P-B-B	10 µg	5	0	Lancet. 2014 Dec 6;384(9959):2046-52
				P-B-B	20 µg	10	0	
				P-B-B	40 µg	8	0	
Live-attenuated-CHIKV[b] (TSI-GSD-218)	USAMRIID	1	-	P	3.1 × 105 PFUs/mL; 0.5 mL IM	30	0	J. Inf. Dis. 1998; 177:634-41
		1	-	P	~2.75 × 104 PFUs/mL; 0.5 mL SQ	19	0	
		1	-	P	3.1 × 105 PFUs/mL; 0.5 mL SQ	42	10	Vaccine 30 (2012) 6713–6720
		1	-	P	~2.75 × 104 PFUs/mL; 0.5 mL SQ	21	1	
		2	-	P	~2.75 × 104 PFUs/mL; 0.5 mL SQ	59	12L[c] 19S[c] 5A[c]	Am J Trop Med Hyg. 2000 Jun;62(6):681-5

[a] P = Prime; B = Boost

[b] Well-tolerated with manageable side effects reported to be caused due to reversion to virulence and not the CHIKV structural proteins

[c] L = Local AEs; S = Systemic AEs, A = Arthralgias

©Oxford University

3.8.2 Clinical Usage of the ChAdOx1 Vector in Vaccine Trials

This will be the first-in-human study employing ChAdOx1 Chik. However, ChAdOx1 vectored vaccines expressing different inserts have previously been used in 161 healthy volunteers taking part in clinical trials conducted by the University of Oxford in the UK (Table 2).

ChAdOx1 encoding the influenza fusion protein NP+M1 has been safely administered to 84 healthy adult volunteers in the UK in two completed clinical trials conducted at The Jenner Institute (FLU004 and FLU005). FLU004 was a phase I, open-label, non-randomised dose escalation study of ChAdOx1 NP+M1. The vaccine was safe, well tolerated and immunogenic, inducing ELISpot responses at all doses. The dose of 2.5 × 1010 vp was chosen for further studies of ChAdOx1 NP+M1 (26).

FLU005 was a multicentre phase I, randomised study to determine the safety and immunogenicity of vaccination regimens employing the candidate influenza vaccines MVA-NP+M1 and ChAdOx1 NP+M1. Sixty-nine healthy adult volunteers have received ChAdOx1 NP+M1 at a dose of 2.5 x 10^{10} vp. Administrations of ChAdOx1 NP+M1 and MVA-NP+M1 vaccines were found to be safe and well-tolerated, in agreement with our previous studies (26-29). The majority of adverse events were mild to moderate in nature and lasted for 1-2 days. The most common local adverse event was arm pain at the site of injection and the most common systemic adverse event was mild fatigue and headache.

TB034 was an open-label, phase I clinical trial in which 42 healthy adult volunteers received the ChAdOx1 viral vector expressing the *Mycobacterium tuberculosis* antigen 85A (ChAdOx1 85A). No major safety concerns associated with ChAdOx1 85A administration have been reported.

ChAdOx1 5T4 has been given in the VANCE01 study which is an ongoing first-in-man open label randomized phase I study to determine the safety and immunogenicity of heterologous prime boost ChAd-MVA vaccination against oncofetal antigen 5T4. To date, 34 participants have received the ChAdOx1 5T4 vaccine at a dose of 2.5 x 10^{10} vp and only mild AEs related to the vaccination have been reported

VAC067 is an ongoing first-in-man study of the ChAdOx1 viral vector expressing dual second generation liver-stage malaria antigens LSA1 and LSAP2 (ChAdOx1 LS2). No significant safety concerns have been reported until this date.

None of the above mentioned clinical trials reported serious adverse events associated with the administration of ChAdOx1, which was shown to have a good safety profile.

.

The ChAdOx1 Chik vaccine most probably forms a structure similar to those of MV-CHIK-022 and VRC-CHKVLP059-00-VP, since it too is comprised solely of structural proteins. Being non-replicative, the vaccine design aims to avoid the AEs of the live-attenuated vaccine.

©Oxford University

Table 1: Clinical Experience with CHIKV structural proteins

CHIKV vaccine construct	Sponsor	Phase	Trial ID	Regimen[a]	Dose	n.	AEs	Reference
Live recombinant measles-virus-based CHIKV vaccine carrying CHIKV C-E3-E2-6K-E1 proteins. (MV-CHIK-022)	Thermis GmbH / Institut Pasteur	1	NCT03028441	P-B	$1{\cdot}5 \times 10^{4}$ TCID50 per 0·05 mL	12	0	Lancet Infect Dis. 2015 May;15(5):519-27.
				P-B	$7{\cdot}5 \times 10^{4}$ TCID50 per 0·25 mL	12	0	
				P-B	$3{\cdot}0 \times 10^{5}$ TCID50 per 1·0 mL	12	0	
Lentiviral Virus-Like-Particle carrying CHIKV E3-E2-6K-E1 proteins. (VRC-CHKVLP059-00-VP)	NIAID	1	NCT01489358	P-B-B	10 µg	5	0	Lancet. 2014 Dec 6;384(9959):2046-52
				P-B-B	20 µg	10	0	
				P-B-B	40 µg	8	0	
Live-attenuated-CHIKV[b] (TSI-GSD-218)	USAMRIID	1	-	P	3.1×10^{5} PFUs/mL; 0.5 mL IM	30	0	J. Inf. Dis. 1998; 177:634-41
		1	-	P	$\sim 2.75 \times 10^{4}$ PFUs/mL; 0.5 mL SQ	19	0	
		1	-	P	3.1×10^{5} PFUs/mL; 0.5 mL SQ	42	10	Vaccine 30 (2012) 6713–6720
		1	-	P	$\sim 2.75 \times 10^{4}$ PFUs/mL; 0.5 mL SQ	21	1	
		2	-	P	$\sim 2.75 \times 10^{4}$ PFUs/mL; 0.5 mL SQ	59	12L[c] 19S[c] 5A[c]	Am J Trop Med Hyg. 2000 Jun;62(6):681-5

[a] P = Prime; B = Boost

[b] Well-tolerated with manageable side effects reported to be caused due to reversion to virulence and not the CHIKV structural proteins

[c] L = Local AEs; S = Systemic AEs, A = Arthralgias

©Oxford University

3.8.2 Clinical Usage of the ChAdOx1 Vector in Vaccine Trials

This will be the first-in-human study employing ChAdOx1 Chik. However, ChAdOx1 vectored vaccines expressing different inserts have previously been used in 161 healthy volunteers taking part in clinical trials conducted by the University of Oxford in the UK (Table 2).

ChAdOx1 encoding the influenza fusion protein NP+M1 has been safely administered to 84 healthy adult volunteers in the UK in two completed clinical trials conducted at The Jenner Institute (FLU004 and FLU005). FLU004 was a phase I, open-label, non-randomised dose escalation study of ChAdOx1 NP+M1. The vaccine was safe, well tolerated and immunogenic, inducing ELISpot responses at all doses. The dose of 2.5×10^{10} vp was chosen for further studies of ChAdOx1 NP+M1 (26).

FLU005 was a multicentre phase I, randomised study to determine the safety and immunogenicity of vaccination regimens employing the candidate influenza vaccines MVA-NP+M1 and ChAdOx1 NP+M1. Sixty-nine healthy adult volunteers have received ChAdOx1 NP+M1 at a dose of 2.5 x 10^{10} vp. Administrations of ChAdOx1 NP+M1 and MVA-NP+M1 vaccines were found to be safe and well-tolerated, in agreement with our previous studies (26-29). The majority of adverse events were mild to moderate in nature and lasted for 1-2 days. The most common local adverse event was arm pain at the site of injection and the most common systemic adverse event was mild fatigue and headache.

TB034 was an open-label, phase I clinical trial in which 42 healthy adult volunteers received the ChAdOx1 viral vector expressing the *Mycobacterium tuberculosis* antigen 85A (ChAdOx1 85A). No major safety concerns associated with ChAdOx1 85A administration have been reported.

ChAdOx1 5T4 has been given in the VANCE01 study which is an ongoing first-in-man open label randomized phase I study to determine the safety and immunogenicity of heterologous prime boost ChAd-MVA vaccination against oncofetal antigen 5T4. To date, 34 participants have received the ChAdOx1 5T4 vaccine at a dose of 2.5 x 10^{10} vp and only mild AEs related to the vaccination have been reported

VAC067 is an ongoing first-in-man study of the ChAdOx1 viral vector expressing dual second generation liver-stage malaria antigens LSA1 and LSAP2 (ChAdOx1 LS2). No significant safety concerns have been reported until this date.

None of the above mentioned clinical trials reported serious adverse events associated with the administration of ChAdOx1, which was shown to have a good safety profile.

©Oxford University

Table 2. Clinical experience with ChAdOx1 viral vector vaccines.

Country	Trial	Vaccine	Age	Route	Dose	Number of Volunteers (Received ChAdOx1)
UK	FLU004	ChAdOx1 NP+M1	18-50	IM	$5x10^8$ vp	3
					$5x10^9$ vp	3
					$2.5x10^{10}$ vp	3
					$5x10^{10}$ vp	6
UK	FLU005	ChAdOx1 NP+M1<br>MVA NP+M1 (week 8)	18-50	IM	$2.5x10^{10}$ vp	12
		ChAdOx1 NP+M1<br>MVA NP+M1 (week 52)	18-50	IM	$2.5x10^{10}$ vp	12
		MVA NP+M1<br>ChAdOx1 NP+M1 (week 8)	18-50	IM	$2.5x10^{10}$ vp	12
		MVA NP+M1<br>ChAdOx1 NP+M1 (week 52)	18-50	IM	$2.5x10^{10}$ vp	9
		ChAdOx1 NP+M1	>50	IM	$2.5x10^{10}$ vp	12
		ChAdOx1 NP+M1<br>MVA NP+M1 (week 8)	>50	IM	$2.5x10^{10}$ vp	12
UK	TB034	ChAdOx1 85A	18-50	IM	$5x10^9$ vp	6
					$2.5x10^{10}$ vp	12
		ChAdOx1 85A<br>MVA85A (week 8)	18-50	IM	$2.5x10^{10}$ vp	12
UK	VANCE01<br>*(ongoing)*	ChAdOx1.5T4<br>MVA.5T4	18 – 75	IM	$2.5x10^{10}$ vp	34 (as of Sep 2017)
UK	VAC067<br>*(ongoing)*	ChAdOx1 LS2	18-45	IM	$5x10^9$ vp	3
					$2.5x10^{10}$ vp	10

3.9 Rationale

CHIKV is an re-emerging outbreak pathogen with approximately 1 million confirmed cases per year worldwide. Autochthonous CHIKV transmission has been reported in over 100 countries in Africa, Asia, the Americas, Oceania and Southern Europe (2)). The long-term risk is that 3.9 billion people in 128 countries could become at risk of infection, the same as that predicted for Dengue virus (30), given transmission is through the same Aedes mosquito species.

©Oxford University

Large outbreaks of CHIKV with high attack rates are common as dense populations increase the infection rate to an estimated 30-75%, with Africa, Asia, Europe, and islands of the Indian and Pacific Oceans affected in recent years. Since 2005, India, Indonesia, the Maldives, Myanmar and Thailand have reported over 1.9 million cases, and in April 2015, over 1.3 million suspected cases of CHIKV had been recorded in the Americas, where it was first detected in late 2013, on islands in the Caribbean. In 2017, outbreaks in the Lazio region of Italy and Provence-Alpes-Côte d'Azur region of southern France have been reported by the WHO.

ChAdOx1 Chik has been developed by The Jenner Institute, University of Oxford, as a prophylactic viral-vectored vaccine against CHIKV. ChAdOx1 Chik employs the structural protein genome of CHIKV (C-E3-E2-6K-E1), inserted into the replication deficient ChAdOx1 vector which possesses adjuvant properties.

Chimpanzee adenovirus vaccine vectors have been safely administered to over 1000 people using a wide range of infectious disease targets including malaria (31), HIV (32), tuberculosis, influenza (26), hepatitis C (33), RSV (34) and, most recently, Ebola (35). ChAdOx1 viral vectored vaccines have shown to be both safe and immunogenic in previous clinical trials in the UK (FLU004, FLU005 and TB034).

The CHIKV structural protein cassette is employed in more complex vaccines currently in Phase II clinical trial (clinicaltrials.gov registration number NCT02861586) , and has been proven to elicit good antibody responses in humans. Single-dose immunisation with ChAdOx1 Chik has however, been shown to elicit higher levels of neutralising antibodies in animal models, with fewer immunisations.

ChAdOx1 Chik has been designed for Low-Middle Income Countries and is an innovation in the CHIKV field in that it offers a cost effective, adjuvant free, single dose CHIKV immunisation to stimulate T cells and the induction of neutralising antibodies in humans. The approach taken should allow significant cost reductions through scale up of manufacture and ultimately licensing of the vaccine could provide an affordable and logistically simple solution to the CHIKV problem.

3.10 Vaccine Development Strategy

This will be a First-in-Human Clinical Trial of ChAdOx1 Chik, which is scheduled to progress to Phase Ib trials in Mexico following its successful completion, meeting the endpoints of safety and immunogenicity. Relevant efficacy studies are also planned, with epidemiology studies currently underway in Mexico to identify a suitable Phase II trial site and statistically significant sample size. Funding for all trials is provided by Innovate UK.

©Oxford University

4. OBJECTIVES AND ENDPOINTS

The number of volunteers has been chosen to generate adequate safety and immunogenicity data to meet these objectives, whilst minimising the number of volunteers exposed to a new vaccination regimen.

4.1 Primary Objective

To assess the safety and tolerability of ChAdOx1 Chik in healthy volunteers.

4.1.1 Primary Outcome Measures

The specific endpoints for safety and reactogenicity will be actively and passively collected data on adverse events.

The following parameters will be assessed for all study groups

- Occurrence of solicited local reactogenicity signs and symptoms for 7 days following the vaccination
- Occurrence of solicited systemic reactogenicity signs and symptoms for 7 days following the vaccination
- Occurrence of unsolicited adverse events for 28 days following the vaccination
- Change from baseline for safety laboratory measures
- Occurrence of serious adverse events during the whole study duration

Volunteers will undergo clinical follow up for adverse events for a further 182 days following completion of the vaccination regimen. SAEs will be collected throughout the study. The duration of follow up reflects the desire to obtain longer term safety data with the first use of ChAdOx1 Chik in humans.

4.2 Secondary Objective

To assess the cellular and humoral immunogenicity of ChAdOx1 Chik in healthy adult volunteers.

4.2.1 Secondary Outcome Measures

Measures of immunogenicity to the ChAdOx1 Chik vaccine may include:

- ELISA to quantify antibodies to CHIKV protein antigens
- Ex vivo ELISpot responses to CHIKV protein antigens

Other exploratory immunology may be carried out in collaboration with other specialist laboratories, including laboratories outside of Europe. This would involve transfer of serum/plasma and/or peripheral blood mononuclear cells (PBMC), but samples would be anonymised. Volunteers will be consented for this.

©Oxford University

5. STUDY OVERVIEW

This is a first-in-human, open-label, dose escalation, phase I clinical trial to assess the safety and immunogenicity of the candidate ChAdOx1 Chik vaccine in healthy volunteers aged 18-50. The vaccine will be administered intramuscularly.

Volunteers will be recruited and vaccinated at the CCVTM, Oxford. There will be 3 study groups and a total of 24 volunteers will be enrolled (Table 3). Staggered enrolment will apply for the first three volunteers within each group. Volunteers will be first recruited into Group 1 and subsequently into Groups 2 and 3 following interim clinical safety reviews (see section 7.4.2). Volunteers will be allocated to a study group by selecting eligible volunteers for enrolment following screening.

5.1 Rationale for Selected Doses

Doses to be administered in this trial have been selected on the basis of clinical experience with the ChAdOx1 adenovirus vector expressing different inserts and similar adenovirus vectored vaccines (eg. ChAd63).

A first-in-human dose escalation study using the ChAdOx1 vector encoding an influenza antigen (FLU004), safely administered ChAdOx1 NP+M1 at doses ranging from 5×10^{8} to 5×10^{10} vp. Subsequent review of the data identified an optimal dose of 2.5×10^{10} vp balancing immunogenicity and reactogenicity. This dose has subsequently been given to over 100 volunteers in numerous larger phase 1 studies at the Jenner Institute (FLU005, TB034 VANCE01 and VAC067) and ChAdOx1 vectored vaccines have thus far demonstrated to be very well tolerated. The vast majority of AEs have been mild-moderate and there have been no SARs reported to date.

Another simian adenovirus vector (ChAd63) has been safely administered at doses up to 2×10^{11} vp with an optimal dose of 5×10^{10} vp, balancing immunogenicity and reactogenicity.

As this is a first-in-human assessment of the Chik antigenic insert, the first dose of ChAdOx1 Chik proposed in this study (5×10^{9} vp) is therefore at least 10 fold less than what this new insert is expected to be tolerated (5×10^{10} vp). Doses will be gradually increased aiming to provide an optimal dose of ChAdOx1 Chik considering the tolerability, reactogenicity and immunogenicity profiles.

©Oxford University

5.2 Study Groups

Table 3. Study Groups

Group	Single Dose ChAdOx1 Chik	Route
Group 1 (n=6)	5 x 10^{9} vp	IM
Group 2 (n=9)	2.5 x 10^{10} vp	IM
Group 3 (n=9)	5 x 10^{10} vp	IM

5.2.1 First Volunteers

Volunteers will be enrolled and doses will be escalated according to the plan outlined below.

The first volunteer in the study will receive 5 x10^{9} vp of ChAdOx1 Chik (group 1). This volunteer will be vaccinated ahead of any other volunteers and the profile of adverse events will be examined after 48h (±24h). Provided there are no safety concerns as assessed by the Chief Investigator (CI) and the Chairman of Local Safety Committee (LSC), another 2 volunteers will be vaccinated at the same dose after at least 48 hours has elapsed following vaccination of the first volunteer and at least 1 hour apart from each other. An independent safety review will be conducted by the Chairman of LSC after vaccination of the first 3 volunteers in group 1. This review will include the results of safety blood tests at day 7 post vaccination and an assessment of the profile of the adverse events reported. The CI and the Chairman of LSC will be asked to provide the decision on whether to proceed with vaccinations of the remaining participants in group 1 (for additional safety and immunology data) and the first volunteer to receive the next incremental dose in group 2. If there are no safety concerns, the remaining volunteers in Group 1 and the first volunteer in group 2 may be vaccinated.

The same procedure will apply for each of the first 3 volunteers enrolled at higher dosage groups and prior to dose escalation (groups 2 and 3).

5.2.2 Duration of study

The total duration of the study will be 26 weeks from the day of enrolment for all volunteers.

5.2.3 Definition of Start and End of Trial

The start of the trial is defined as the date of the first vaccination of the first volunteer. The end of the trial is the date of the last visit of the last volunteer.

©Oxford University

5.3 Potential Risks for volunteers

The potential risk to participants is considered as low. The potential risks are those associated with phlebotomy and vaccination. In general, recombinant adenoviral vectors are safe. Similar vaccines encoding different antigens have been given to several thousand volunteers (including children) with a good safety profile.

Phlebotomy:

The maximum volume of blood drawn over the study period (approximately 285mL) should not compromise these otherwise healthy volunteers. There may be minor bruising, local tenderness or pre-syncopal symptoms associated with venepuncture, which will not be documented as AEs if they occur.

Vaccination:

ChAdOx1 Chik has not been used in humans before and therefore will be initially administered at the lower dose of 5×10^9 vp before progressing to the higher doses of 2.5×10^{10} and 5×10^{10} in Groups 2 and 3. Expected risks from vaccination include local effects such as pain, redness, warmth, swelling, tenderness or itching. Systemic reactions that could potentially occur following immunisation with a recombinant adenovirus vaccine include a flu-like illness with feverishness, fatigue, malaise, arthralgia, myalgia and headache.

As with any vaccine, Guillain-Barré syndrome or immune-mediated reactions that can lead to organ damage may occur, but this should be extremely rare. Serious allergic reactions including anaphylaxis could also occur and for this reason volunteers will be vaccinated in a clinical area where Advanced Life Support trained physicians, equipment and drugs are immediately available for the management of any serious adverse reactions (SAR).

5.4 Known Potential Benefits

Volunteers will not benefit directly from participation in this study. However, it is hoped that the information gained from this study will contribute to the development of a safe and effective CHIKV vaccine regime. The only benefits for participants would be information about their general health status.

©Oxford University

6. RECRUITMENT AND WITHDRAWAL OF TRIAL VOLUNTEERS

6.1 Volunteers

Volunteers may be recruited by use of an advertisement +/- registration form formally approved by the ethics committee(s) and distributed or posted in the following places:

- In public places, including buses and trains, with the agreement of the owner/proprietor.
- In newspapers or other literature for circulation.
- On radio via announcements.
- On a website or social media site operated by our group or with the agreement of the owner or operator (including on-line recruitment through our web-site).
- By e-mail distribution to a group or list only with the express agreement of the network administrator or with equivalent authorisation.
- By email distribution to individuals who have already expressed an interest in taking part in any clinical trial at the Oxford Vaccine Centre.
- On stalls or stands at exhibitions or fairs.
- Via presentations (e.g. presentations at lectures or invited seminars).
- Direct mail-out: This will involve obtaining names and addresses of adults via the most recent Electoral Roll. The contact details of individuals who have indicated that they do not wish to receive postal mail-shots would be removed prior to the investigators being given this information. The company providing this service is registered under the Data Protection Act 1998. Investigators would not be given dates of birth or ages of individuals but the list supplied would only contain names of those aged between 18-50 years (as per the inclusion criteria).
- Oxford Vaccine Centre databases: We may contact individuals from databases of groups within the CCVTM (including the Oxford Vaccine Centre database) of previous trial participants who have expressed an interest in receiving information about all future studies for which they may be eligible.

6.2 Informed consent

All volunteers will sign and date the informed consent form before any study specific procedures are performed. The information sheet will be made available to the volunteer at least 24 hours prior to the screening visit. At the screening visit, the volunteer will be fully informed of all aspects of the trial, the potential risks and their obligations. The following general principles will be emphasised:

- Participation in the study is entirely voluntary
- Refusal to participate involves no penalty or loss of medical benefits
- The volunteer may withdraw from the study at any time
- The volunteer is free to ask questions at any time to allow him or her to understand the purpose of the study and the procedures involved

©Oxford University

- The study involves research of an investigational vaccine
- There is no direct benefit from participating
- The volunteer's GP will be contacted to corroborate their medical history
- The volunteer's blood samples taken as part of the study will be stored indefinitely and samples may be sent outside of the UK and Europe to laboratories in collaboration with the University of Oxford. These will be anonymised.

The aims of the study and all tests to be carried out will be explained. The volunteer will be given the opportunity to ask about details of the trial, and will then have time to consider whether or not to participate. If they do decide to participate, they will sign and date two copies of the consent form, one for them to take away and keep, and one to be stored in the case report form (CRF) – this is a paper or electronic document used to collect data relating to a particular volunteer. These forms will also be signed and dated by the Investigator.

6.3 Inclusion and exclusion criteria

This study will be conducted in healthy adults, who meet the following inclusion and exclusion criteria:

6.3.1 Inclusion Criteria

The volunteer must satisfy all the following criteria to be eligible for the study:

1. Healthy adults aged 18 to 50 years
2. Able and willing (in the Investigator's opinion) to comply with all study requirements
3. Willing to allow the investigators to discuss the volunteer's medical history with their General Practitioner
4. For females only, willingness to practice continuous effective contraception (see below) during the study and a negative pregnancy test on the day(s) of screening and vaccination
5. Agreement to refrain from blood donation during the course of the study
6. Provide written informed consent

6.3.2 Exclusion Criteria

The volunteer may not enter the study if any of the following apply:

1. Participation in another research study involving receipt of an investigational product in the 30 days preceding enrolment, or planned use during the study period
2. Prior receipt of an investigational vaccine likely to impact on interpretation of the trial data (e.g. Adenovirus vectored vaccine).
3. Administration of immunoglobulins and/or any blood products within the three months preceding the planned administration of the vaccine candidate

©Oxford University

4. Any confirmed or suspected immunosuppressive or immunodeficient state, including HIV infection; asplenia; recurrent, severe infections and chronic (more than 14 days) immunosuppressant medication within the past 6 months (inhaled and topical steroids are allowed)
5. History of allergic disease or reactions likely to be exacerbated by any component of the vaccine
6. Any history of hereditary angioedema, acquired angioedema, or idiopathic angioedema.
7. Any history of anaphylaxis in relation to vaccination
8. Pregnancy, lactation or willingness/intention to become pregnant during the study
9. History of cancer (except basal cell carcinoma of the skin and cervical carcinoma in situ)
10. History of serious psychiatric condition likely to affect participation in the study
11. Bleeding disorder (eg. factor deficiency, coagulopathy or platelet disorder), or prior history of significant bleeding or bruising following IM injections or venepuncture
12. Any other serious chronic illness requiring hospital specialist supervision
13. Suspected or known current alcohol abuse as defined by an alcohol intake of greater than 42 units every week
14. Suspected or known injecting drug abuse in the 5 years preceding enrolment
15. Seropositive for hepatitis B surface antigen (HBsAg)
16. Seropositive for hepatitis C virus (antibodies to HCV)
17. Any clinically significant abnormal finding on screening biochemistry or haematology blood tests or urinalysis
18. Any other significant disease, disorder or finding which may significantly increase the risk to the volunteer because of participation in the study, affect the ability of the volunteer to participate in the study or impair interpretation of the study data
19. Inability of the study team to contact the volunteer's GP to confirm medical history and safety to participate
20. Prior exposure to CHIKV (serology will be requested at the discretion of the investigator)
21. Travel to a CHIKV endemic region throughout the duration of the participants enrolment in the Study and within the preceding 30 days.

6.3.3 Effective contraception for female volunteers

Female volunteers are required to use an effective form of contraception during the course of the study (i.e until their last follow up visit). As this is a Phase I, first-in-human, study there is no information about the effect of this vaccine on a foetus. Male subjects with female partners of child-bearing potential are not required to use barrier methods for the purposes of contraception whilst taking part in this study as the risk of excretion of the vaccine is negligible.

©Oxford University

Acceptable forms of contraception for female volunteers include:

- Established use of oral, injected or implanted hormonal methods of contraception.
- Placement of an intrauterine device (IUD) or intrauterine system (IUS).
- Total abdominal hysterectomy
- Barrier methods of contraception (condom or occlusive cap with spermicide)
- Male sterilisation, if the vasectomised partner is the sole partner for the subject.
- True abstinence: when this is in line with the preferred and usual lifestyle of the subject. Periodic abstinence (e.g. calendar, ovulation, symptothermal, post-ovulation methods), declaration of abstinence for the duration of exposure to IMP, and withdrawal are not acceptable methods of contraception

6.3.4 Prevention of 'Over Volunteering'

Volunteers will be excluded from the study if they are concurrently involved in another trial. In order to check this, volunteers will be asked to provide their National Insurance or Passport number (if they are not entitled to a NI number) and will be registered on a national database of participants in clinical trials (*www.tops.org.uk*).

6.3.5 Criteria for postponement of vaccination

The following events constitute contraindications to administration of the vaccine at that point in time; if any one of these events occurs at the time scheduled for vaccination, the subject may be vaccinated at a later date, or withdrawn at the discretion of the Investigator.

- Acute disease at the time of vaccination. Acute disease is defined as the presence of a moderate or severe illness with or without fever. All vaccines can be administered to persons with a minor illness such as diarrhoea, mild upper respiratory infection with or without low-grade febrile illness, i.e. temperature of ≤37.5°C/99.5°F.
- Temperature of >37.5°C (99.5°F) at the time of vaccination.

6.3.6 Withdrawal of Volunteers

In accordance with the principles of the current revision of the Declaration of Helsinki and any other applicable regulations, a volunteer has the right to withdraw from the study at any time and for any reason, and is not obliged to give his or her reasons for doing so. The Investigator may withdraw the volunteer at any time in the interests of the volunteer's health and well-being. In addition the volunteer may withdraw/be withdrawn for any of the following reasons:

- Administrative decision by the Investigator.
- Ineligibility (either arising during the study or retrospectively, having been overlooked at screening).
- Significant protocol deviation.

©Oxford University

- Volunteer non-compliance with study requirements.
- An AE, which requires discontinuation of the study involvement or results in inability to continue to comply with study procedures.

The reason for withdrawal will be recorded in the CRF. If withdrawal is due to an AE, appropriate follow-up visits or medical care will be arranged, with the agreement of the volunteer, until the AE has resolved, stabilised or a non-trial related causality has been assigned. Any volunteer who is withdrawn from the study may be replaced, if that is possible within the specified time frame. The Chairman of LSC may recommend withdrawal of volunteers.

Any volunteer who fails to attend for two or more follow-up visits during the study will be deemed to have withdrawn from the study.

If a volunteer withdraws from the study, blood samples collected before their withdrawal from the trial will be used/ stored unless the volunteer specifically requests otherwise.

In all cases of subject withdrawal, excepting those of complete consent withdrawal, long-term safety data collection, including some procedures such as safety bloods, will continue as appropriate if subjects have received one or more vaccine doses.

6.4 Compliance with Dosing Regime

All doses in this vaccine study will be administered by the Investigator and recorded in the CRF. The study medication will be at no time in the possession of the volunteer and compliance will, therefore, not be an issue.

6.5 Pregnancy

Should a volunteer become pregnant during the trial, she will be followed up as other volunteers and in addition will be followed until pregnancy outcome. We will not routinely perform venepuncture in a pregnant volunteer.

©Oxford University

7. CLINICAL PROCEDURES

This section describes the clinical procedures for evaluating study participants and follow-up after administration of study vaccine.

7.1 Study procedures

All volunteers will have the same schedule of clinic attendances and procedures as indicated in the schedules of attendance (Table 5). All subjects will receive the ChAdOx1 Chik vaccine, and undergo follow-up for a total of 26 weeks. The total volume of blood donated during the study will be 289mL. Additional visits or procedures may be performed at the discretion of the investigators, e.g., further medical history and physical examination, urine microscopy in the event of positive urinalysis or additional blood tests if clinically relevant.

7.2 Observations

Pulse, blood pressure and temperature will be measured at the time-points indicated in the schedule of procedures and may also be measured as part of a physical examination if indicated at other time-points.

7.3 Blood Tests and Urinalysis

Blood will be drawn for the following laboratory tests and processed:

1. At Oxford University Hospitals' NHS Trust using NHS standard procedures:

- **Haematology;** Full Blood Count
- **Biochemistry;** Sodium, Potassium, Urea, Creatinine, Albumin, Liver Function Tests (ALT, ALP, Bilirubin)
- **Diagnostic serology;** HBsAg, HCV antibodies, HIV antibodies (specific consent will be gained prior to testing blood for these blood-borne viruses)
- **Immunology;** Human Leukocyte Antigen (HLA) typing

Additional safety blood tests may be performed if clinically relevant at the discretion of the medically qualified investigators. These generally include, but are not limited to AST, GGT and a coagulation screen.

2. At University of Oxford research laboratories:

- **Exploratory Immunology;** Immunogenicity will be assessed by a variety of immunological assays. This may include antibodies to CHIKV and other related viruses, ex vivo ELISpot assays for interferon gamma and flow cytometry assays, functional antibody assays and B cell analyses. Other exploratory immunological assays including cytokine analysis and other antibody assays, DNA analysis of genetic

©Oxford University

polymorphisms potentially relevant to vaccine immunogenicity and gene expression studies amongst others may be performed at the discretion of the Investigators.

3. **Urinalysis;** Urine will be tested for protein, blood and glucose at screening. For female volunteers only, urine will be tested for beta-human chorionic gonadotrophin (β-HCG) at screening and immediately prior to each vaccination.

Collaboration with other specialist laboratories in the UK, Europe and outside of Europe for further exploratory immunological tests may occur. This would involve the transfer of serum or plasma and/or PBMC to these laboratories, but these would remain anonymised. Informed consent for this will be gained from volunteers. Immunological assays will be conducted according to local SOPs.

Subjects will be informed that there may be leftover samples of their blood (after all testing for this study is completed), and that such samples may be stored indefinitely in the Oxford Vaccine Center Biobank for possible future research (exploratory immunology), including human DNA and RNA analyses to search for correlates of vaccine immunogenicity and efficacy. Subjects will be able to decide if they will permit such future use of any leftover samples. If a subject elects not to permit this, all of that subject's leftover samples will be discarded after the required period of storage to meet Good Clinical Practice (GCP) and regulatory requirements.

7.4 Study visits

The study visits and procedures will be undertaken by one of the clinical trials team. The procedures to be included in each visit are documented in the schedule of attendances (Table 5). Each visit is assigned a time-point and a window period, within which the visit will be conducted.

7.4.1 Screening visit

All potential volunteers will have a screening visit, which may take place up to 90 days prior to vaccination. Informed consent will be taken before screening, as described in section 6.2. If consent is obtained, the screening procedures indicated in the schedule of attendances will be undertaken. To avoid unnecessary additional venepuncture, if the appropriate blood test results for screening are available for the same volunteer from a screening visit for another Jenner Institute Clinical Trials group vaccine study, these results may be used for assessing eligibility (provided the results date is within the 3 months preceding enrolment in CHIK001).

The subject's general practitioner will be contacted with the written permission of the subject after satisfactory screening as notification that the subject has volunteered for the

©Oxford University

study and to ascertain any significant medical history. During the screening the volunteers will be asked to provide their National Insurance or passport number so that this can be entered on to a national database which helps prevent volunteers from participating in more than one clinical trial simultaneously or over-volunteering for clinical trials (www.tops.org.uk).

Abnormal clinical findings from the urinalysis or blood tests at screening will be assessed by the lead clinician according to the relevant SOP. Abnormal blood tests following screening will be assessed according to site-specific laboratory adverse event grading tables which are filed in the trial master file (TMF) or the Investigator Site File (ISF). Any abnormal test result deemed clinically significant may be repeated to ensure it is not a single occurrence. If an abnormal finding is deemed to be clinically significant, the volunteer will be informed and appropriate medical care arranged with the permission of the volunteer.

The eligibility of the volunteer will be reviewed at the end of the screening visit and again when all results from the screening visit have been considered. Decisions to exclude the volunteer from enrolling in the trial or to withdraw a volunteer from the trial will be at the discretion of the Investigator. If eligible, a day 0 visit will be scheduled for the volunteer to receive the vaccine.

7.4.2 Day 0: Enrolment and Vaccination Visit

Volunteers will not be considered enrolled in the study until they have received a vaccine. Before vaccination, the eligibility of the volunteer will be reviewed. Pulse, blood pressure and temperature will be observed and if necessary, a medical history and physical examination may be undertaken to determine need to postpone vaccination depending on criteria listed in section 6.3.5. Vaccinations will be administered as described below.

7.4.2.1 Vaccinations

Before each vaccination, the on-going eligibility of the volunteer will be reviewed. All vaccines will be administered intramuscularly according to SOP VC002 Vaccination as described below in section 8.4 and the vaccine dilution SOP when required. The injection site will be covered with a sterile dressing and the volunteer will stay in the CCVTM for observation, in case of immediate adverse events. Observations will be taken 30 minutes after vaccination (+/- 5 minutes) and the sterile dressing removed and injection site inspected. Observations will also be taken at 60 minutes (+/- 10 minutes), before the volunteer leaves. An oral thermometer, tape measure and diary card (paper or electronic) will be given to each volunteer, with instructions on use, along with the emergency 24 hour telephone number to contact the on-call study physician if needed.

Diary cards will collect information on the timing and severity of the following solicited AEs:

Table 4. Solicited AEs as collected on post vaccination diary cards

©Oxford University

Local solicited AEs	Systemic solicited AEs
Pain	Fever
Redness	Feverishness
Warmth	Joint pains
Itch	Muscle pains
	Fatigue
	Headache
	Nausea
	Malaise

Volunteers will be instructed on how to self-assess the severity of these AEs. There will also be space on the diary card to self-document unsolicited AEs, and whether medication was taken to relieve the symptoms.

7.4.2.2 Sequence of Enrolment and Vaccination of Volunteers

For safety reasons, the first volunteer in Group 1 will be vaccinated ahead of any other volunteers and the profile of adverse events will be reviewed after 48 hours (±24h) post vaccination. Provided there are no safety concerns, as assessed by the CI and the Chairman of LSC, another 2 volunteers will be vaccinated at the same dose after at least 48 hours has elapsed following the first volunteer being vaccinated and at least 1 hour apart from each other. An independent safety review will be conducted by the Chairman of LSC after vaccination of the first three volunteers. This review will include an assessment of the profile of adverse events and the results of safety blood tests at day 7 post vaccination. The CI and the Chairman of LSC will be asked to provide the decision on whether to proceed with vaccinations of the remaining participants in group 1 and the first volunteer to receive the next incremental dose in group 2. If there are no safety concerns, the remaining volunteers in Group 1 and the first volunteer in group 2 may be vaccinated.

Enrolment of the first volunteer in Group 2 will only proceed if the CI and Chairman of LSC assess the data from the first three vaccinees in Group 1 as indicating that it is safe to do so. The first subject in Group 2 will be vaccinated alone, and a 48 hour gap allowed before vaccinating further subjects in this group. Provided there are no safety concerns, as assessed by the CI and the Chairman of LSC, another 2 volunteers will be vaccinated at the same dose after at least 48 hours has elapsed following the first volunteer being vaccinated and at least 1 hour apart from each other. An independent safety review will be conducted by the Chairman of LSC after vaccination of the first three volunteers. This review will include an assessment of the profile of adverse events and the results of safety blood tests at day 7 post vaccination. The CI and the Chairman of LSC will be asked to provide the

©Oxford University

decision on whether to proceed with vaccinations of the remaining participants in group 2 and the first volunteer to receive the next incremental dose in group 3. If there are no safety concerns, the remaining volunteers in Group 2 and the first volunteer in group 3 may be vaccinated.

Enrolment of the first volunteer in Group 3 will only proceed if the CI and Chairman of LSC assess the data from the first three vaccinees in Group 2 as indicating that it is safe to do so. The first subject in Group 3 will be vaccinated alone, and a 48 hour gap allowed before vaccinating further subjects in this group. Provided there are no safety concerns, as assessed by the CI and the Chairman of LSC, another 2 volunteers will be vaccinated at the same dose after at least 48 hours has elapsed following the first volunteer being vaccinated and at least 1 hour apart from each other. An independent safety review will be conducted by the Chairman of LSC after vaccination of the first three volunteers. This review will include an assessment of the profile of adverse events and the results of safety blood tests at day 7 post vaccination. The CI and the Chairman of LSC will be asked to provide the decision on whether to proceed with vaccinations of the remaining participants in group 3. If there are no safety concerns, the remaining volunteers in Group 3 may be vaccinated.

7.4.3 Subsequent visits: days 2, 7, 14, 28, 56 and 182.

Follow-up visits will take place 48 hours (±24h), 7 days (± 2 days), 14 days (± 3 days), 28 days (± 3 days), 56 days (± 7 days) and 182 (± 14 days) after vaccination. Volunteers will be assessed for local and systemic adverse events, interim history, physical examination, review of diary cards (paper or electronic) and blood tests at these time points as detailed in the schedule of attendances. Blood will also be taken for exploratory immunology purposes.

If volunteers experience adverse events (laboratory or clinical), which the investigator (physician), CI and/or Chairman of LSC determine necessary for further close observation, the volunteer may be admitted to an NHS hospital for observation and further medical management under the care of the Consultant on call.

©Oxford University

Table 5. Schedule of attendances

Attendance Number	1^S	2	3	4	5	6	7	8
Timeline** (days)	≤ 90	0	2	7	14	28	56	182
Time window (days)			±1	±2	±3	±3	±7	±14
Informed Consent	X							
Review contraindications, inclusion and exclusion criteria	X	X						
Vaccination		X						
Vital signs^	X	X	X	X	X	X	X	X
Ascertainment of adverse events		X	X	X	X	X	X	X
Diary cards provided		X						
Diary cards collected						X		
Medical History, Physical Examination	X	(X)	(X)	(X)	(X)	(X)	(X)	(X)
Biochemistry$, Haematology (mL)	5	5	5	5		5		
Exploratory immunology£ (mL)	5	50			50	50	50	50
Urinalysis	X							
Urinary β–HCG (women only)	X	X						
HLA typing (mL)		4						
HBsAg, HCV Ab, HIV serology (mL)	5							
Blood volume per visit	15	59	5	5	50	55	50	50
Cumulative blood volume%	15	74	79	84	134	189	239	289

S = screening visit; (X) = if considered necessary ^ = Vital signs includes pulse, blood pressure and temperature; $ = Biochemistry will include Sodium, Potassium, Urea, Creatinine, Albumin and Liver function tests. £ = Exploratory immunology includes antibodies to CHIKV and related viruses, ex vivo interferon-gamma ELISpot responses to CHIKV proteins. Serology and other immunology tests for CHIKV and related viruses may be perfomed at screening to help determine prior CHIKV exposure in certain volunteers

** Timeline is approximate only. Exact timings of visits relate to the day on enrolment, ie, each visit must occur at indicated number of days after enrolment ± time window.

% Cumulative blood volume for Oxford volunteers if blood taken as per schedule, and excluding any repeat safety blood test that may be necessary.

©Oxford University

8. INVESTIGATIONAL PRODUCTS

The following vaccinations will be given in this study:

1. ChAdOx1 Chik 5 x 10^9vp
2. ChAdOx1 Chik 2.5 x 10^{10}vp
3. ChAdOx1 Chik 5 x 10^{10}vp

8.1. Manufacturing and Presentation

8.1.1 Description of ChAdOx1 Chik

ChAdOx1 Chik vaccine consists of the replication-deficient simian adenovirus vector ChAdOx1, containing the whole structural gene cassette for the precursors of viral structural proteins (C-E3-E2-6K-E1) of the CHIKV , expressed from the strong CMV IE promoter.

8.1.2. ChAdOx1 Chik formulation and packaging

ChAdOx1 Chik is manufactured in formulation buffer to a target concentration of ≥1.1 x 10^{11} vp/mL. The drug product is filled into 2mL glass vials with a 13 mm grey bromobutyl rubber freeze-dry stopper (CE Marked, supplied by Adelphi Tubes) and a 13 mm aluminium seal. The nitrogen filled vials are supplied sterile. The containers and closures are tested for compliance with defined specifications. The vials are made from Ph Eur Type 1 glass.

8.2 Supply

ChAdOx1 Chik has been formulated and vialed under Good Manufacturing Practice conditions at the Clinical Biomanufacturing Facility (CBF), University of Oxford. At the CBF the vaccine will be certified and labelled for the trial by a Qualified Person (QP) before transfer to the clinical site.

8.3 Storage

The vaccine is stored at nominal -80°C in a locked freezer, at the clinical site. All movements of the study vaccines will be documented in accordance with existing standard operating procedure (SOP). Vaccine accountability, storage, shipment and handling will be in accordance with relevant SOPs and forms.

8.4 Administration of Investigational Medicinal Products

On vaccination day, ChAdOx1 Chik will be allowed to thaw to room temperature and will be administered within 1 hour of removal from the freezer. The vaccine will be administered intramuscularly into the deltoid of the non-dominant arm (preferably). All volunteers will be observed in the unit for 1 hour (±10 minutes) after vaccination. During administration of the investigational products, Advanced Life Support drugs and resuscitation equipment will

©Oxford University

be immediately available for the management of anaphylaxis. Vaccination will be performed and the IMPs handled according to the relevant SOPs.

8.5 Minimising environmental contamination with genetically modified organisms (GMO)

The study will be performed in accordance with UK Genetically Modified Organisms (Contained Use) Regulations (2014). In order to minimise dissemination of the recombinant vectored vaccine virus into the environment, inoculation sites will be covered with a dressing after immunisation. This should absorb any virus that may leak out through the needle track. The dressing will be removed from the injection site after 30 minutes (+15/- 5 minutes) and will be disposed as GMO waste by autoclaving.

©Oxford University

9. ASSESSMENT OF SAFETY

Safety will be assessed by the frequency, incidence and nature of adverse events and serious adverse events arising during the study.

9.1 Definitions

9.1.1 Adverse Event (AE)

An AE is any untoward medical occurrence in a volunteer, which may occur during or after administration of an Investigational Medicinal Product (IMP) and does not necessarily have a causal relationship with the intervention. An AE can therefore be any unfavourable and unintended sign (including an abnormal laboratory finding), symptom or disease temporally associated with the study intervention, whether or not considered related to the study intervention.

9.1.2 Adverse Reaction (AR)

An AR is any untoward or unintended response to an IMP. This means that a causal relationship between the IMP and an AE is at least a reasonable possibility, i.e., the relationship cannot be ruled out. All cases judged by the reporting medical Investigator as having a reasonable suspected causal relationship to an IMP (i.e. possibly, probably or definitely related to an IMP) will qualify as adverse reactions.

9.1.3 Unexpected Adverse Reaction

An adverse reaction, the nature or severity of which is not consistent with the applicable product information (e.g., IB for an unapproved IMP).

9.1.4 Serious Adverse Event (SAE)

An SAE is an AE that results in any of the following outcomes, whether or not considered related to the study intervention.

- Death
- Life-threatening event (i.e., the volunteer was, in the view of the Investigator, at immediate risk of death from the event that occurred). This does not include an AE that, if it occurred in a more severe form, might have caused death.
- Persistent or significant disability or incapacity (i.e., substantial disruption of one's ability to carry out normal life functions).
- Hospitalisation, regardless of length of stay, even if it is a precautionary measure for continued observation. Hospitalisation (including inpatient or outpatient hospitalisation for an elective procedure) for a pre-existing condition that has not worsened unexpectedly does not constitute a serious AE.

©Oxford University

- An important medical event (that may not cause death, be life threatening, or require hospitalisation) that may, based upon appropriate medical judgment, jeopardise the volunteer and/or require medical or surgical intervention to prevent one of the outcomes listed above. Examples of such medical events include allergic reaction requiring intensive treatment in an emergency room or clinic, blood dyscrasias, or convulsions that do not result in inpatient hospitalisation.
- Congenital anomaly or birth defect.

9.1.5 Serious Adverse Reaction (SAR)

An adverse event (expected or unexpected) that is both serious and, in the opinion of the reporting Investigator or Sponsors, believed to be possibly, probably or definitely due to an IMP or any other study treatments, based on the information provided.

9.1.6 Suspected Unexpected Serious Adverse Reaction (SUSAR)

A serious adverse reaction, the nature and severity of which is not consistent with the information about the medicinal product in question set out in the IB or Summary of Product Characteristics (SmPC).

9.2 Foreseeable Adverse Reactions:

The foreseeable ARs following vaccination with ChAdOx1 Chik include injection site pain, erythema, warmth, swelling, pruritus, myalgia, arthralgia, headache, fatigue, fever, feverishness, malaise and nausea.

9.3 Expected Serious Adverse Events

No serious adverse events are expected in this study.

9.4 Causality Assessment

For every AE, an assessment of the relationship of the event to the administration of the vaccine will be undertaken by the CI-delegated clinician. An intervention-related AE refers to an AE for which there is a probable or definite relationship to administration of a vaccine. An interpretation of the causal relationship of the intervention to the AE in question will be made, based on the type of event; the relationship of the event to the time of vaccine administration; and the known biology of the vaccine therapy (Table 6). Alternative causes of the AE, such as the natural history of pre-existing medical conditions, concomitant therapy, other risk factors and the temporal relationship of the event to vaccination will be considered and investigated. Causality assessment will take place during planned safety reviews, interim analyses (e.g. if a holding or stopping rule is activated) and at the final safety analysis, except for SAEs, which should be assigned by the reporting investigator.

©Oxford University

Table 6. Guidelines for assessing the relationship of vaccine administration to an AE.

0	**No Relationship**	No temporal relationship to study product ***and***<br>Alternate aetiology (clinical state, environmental or other interventions); ***and***<br>Does not follow known pattern of response to study product
1	**Unlikely**	Unlikely temporal relationship to study product ***and***<br>Alternate aetiology likely (clinical state, environmental or other interventions) ***and***<br>Does not follow known typical or plausible pattern of response to study product.
2	**Possible**	Reasonable temporal relationship to study product; ***or***<br>Event not readily produced by clinical state, environmental or other interventions; ***or***<br>Similar pattern of response to that seen with other vaccines
3	**Probable**	Reasonable temporal relationship to study product; ***and***<br>Event not readily produced by clinical state, environment, or other interventions ***or***<br>Known pattern of response seen with other vaccines
4	**Definite**	Reasonable temporal relationship to study product; ***and***<br>Event not readily produced by clinical state, environment, or other interventions; ***and***<br>Known pattern of response seen with other vaccines

9.5 Reporting Procedures for All Adverse Events (see SOP VC027)

All local and systemic AEs occurring in the 28 days following each vaccination observed by the Investigator or reported by the volunteer, whether or not attributed to study medication, will be recorded (excluding those expected consequences from venepuncture, described in section 5.3). Recording and reporting of all AEs will take place as detailed in SOP VC027. All AEs that result in a volunteer's withdrawal from the study will be followed up until a satisfactory resolution occurs, or until a non-study related causality is assigned (if the volunteer consents to this). Serious adverse events (SAEs) will be collected throughout the entire trial period.

9.5.1 Reporting Procedures for Serious AEs (see SOP OVC005 Safety Reporting)

In order to comply with current regulations on serious adverse event reporting to regulatory authorities, the event will be documented accurately and notification deadlines respected.

©Oxford University

SAEs will be reported on the SAE forms to members of the study team immediately the Investigators become aware of their occurrence, as described in SOP OVC005. Copies of all reports will be forwarded for review to the Chief Investigator (as the Sponsor's representative) within 24 hours of the Investigator being aware of the suspected SAE. The Chairman of LSC will be notified of SAEs that are deemed possibly, probably or definitely related to study interventions; the Chairman of LSC will be notified immediately (within 24 hours) of the Investigators' being aware of their occurrence. SAEs will not normally be reported immediately to the ethical committee(s) unless there is a clinically important increase in occurrence rate, an unexpected outcome, or a new event that is likely to affect safety of trial volunteers, at the discretion of the Chief Investigator and/or Chairman of LSC. In addition to the expedited reporting above, the Investigator shall include all SAEs in the annual Development Safety Update Report (DSUR) report.

9.5.2 Reporting Procedures for SUSARS

The Chief Investigator will report all SUSARs to the MHRA and ethical committee(s) within required timelines (15 days for all SUSARs, unless life threatening in which case 7 days, with a final report within a further 8 days (total 15). The Chief Investigator will also inform all Investigators concerned of relevant information about SUSARs that could adversely affect the safety of participants. All SUSARs and deaths occurring during the study will be reported to the Sponsor. For all deaths, available autopsy reports and relevant medical reports will be made available for reporting to the relevant authorities.

9.5.3 Development Safety Update Report

A Development Safety Update Report (DSUR) will be submitted by the Sponsor to the competent authority and ethical committee on the anniversary of the first approval date from the regulatory authority for each IMP.

9.6 Assessment of severity

The severity of clinical and laboratory adverse events will be assessed according to the scales in Tables 7-9, also described in the SOP VC027.

©Oxford University

Table 7. Severity grading criteria for local adverse events.

Adverse Event	Grade	Intensity
Erythema at injection site*	1	>3 - ≤50 mm
	2	>50 - ≤100 mm
	3	>100 mm
Swelling at injection site	1	>3 - ≤20 mm
	2	>20 - ≤50 mm
	3	>50 mm
Ulceration/necrosis of skin at injection site	1	-
	2	-
	3	Any

*erythema or swelling ≤3mm is an expected consequence of skin puncture and will therefore not be considered an adverse event.

Table 8. Severity grading criteria for physical observations

	**Grade 1 (mild)**	**Grade 2 (moderate)**	**Grade 3 (severe)**
Fever (oral)	37.6°C - 38.0°C	38.1°C – 39.0°C	>39.0°C
Tachycardia (bpm)*	101 - 115	116 – 130	>130
Bradycardia (bpm)**	50 – 54	40 – 49	<40
Systolic hypertension (mmHg)	141 - 159	160 – 179	≥180
Systolic hypotension (mmHg)***	85 - 89	80 – 84	<80
Diastolic hypertension (mmHg)	91 - 99	100 – 109	≥110

*Taken after ≥10 minutes at rest

**Use clinical judgement when characterising bradycardia among some healthy subject populations, for example, conditioned athletes.

***Only if symptomatic (e.g. dizzy/ light-headed)

©Oxford University

Table 9. Severity grading criteria for local and systemic AEs.

**GRADE 0**	None: Symptom not experienced
**GRADE 1**	Mild: Short-lived or mild symptoms; medication may be required. No limitation to usual activity
**GRADE 2**	Moderate: Mild to moderate limitation in usual activity. Medication may be required.
**GRADE 3**	Severe: Considerable limitation in activity. Medication or medical attention required.

9.7 Procedures to be followed in the event of abnormal findings

Laboratory parameters for inclusion/exclusion in the trial will be considered on an individual basis, with investigator discretion for interpretation of results and the need for repeated tests. Laboratory adverse events will be assessed using the tables as detailed in SOP VC027. Abnormal clinical findings from medical history, examination or blood tests will be assessed as to their clinical significance throughout the trial. If a test is deemed clinically significant, it may be repeated, to ensure it is not a single occurrence. If a test remains clinically significant, the volunteer will be informed and appropriate medical care arranged as appropriate and with the permission of the volunteer. Decisions to exclude the volunteer from enrolling in the trial or to withdraw a volunteer from the trial will be at the discretion of the Investigator.

9.8 Local Safety Committee

A Local Safety Committee (LSC) will be appointed to provide real-time safety oversight. The LSC will review SAEs deemed possibly, probably or definitely related to study interventions. The LSC will be notified within 24 hours of the Investigators' being aware of their occurrence. The LSC has the power to place the study on hold if deemed necessary following a study intervention-related SAE. At the time of writing the LSC will be chaired by Dr Brian Angus, a Clinical Tutor in Medicine, Honorary Consultant Physician and Director, Centre for Tropical Medicine at the University of Oxford, . There will be a minimum of two other appropriately qualified committee members.

The chair of the LSC may be contacted for advice and independent review by the Investigator or trial Sponsor in the following situations:

- Following any SAE deemed to be possibly, probably, or definitely related to a study intervention.
- Any other situation where the Investigator or trial Sponsor feels independent advice or review is important.

©Oxford University

9.8.1 Interim Safety Reviews

Interim safety reviews with the Chairman of the LSC are scheduled during the enrolment of the first volunteers in each group and prior to dose escalations, as outlined in section 7.4.2.2.

The safety profile of the IMP will be assessed on an on-going basis by the Investigators with communication to the LSC as necessary. The Chief Investigator and relevant Investigators (as per the trial delegation log) will also review safety issues and SAEs as they arise.

9.9 Safety Stopping/Holding Rules

Safety holding rules have been developed considering the fact that this is a first-in-human dose escalation study.

'Solicited adverse events' are those listed as foreseeable adverse events in section 9.3 of the protocol, occurring within the first 7 days after vaccination (day of vaccination and six subsequent days). 'Unsolicited adverse events' are adverse events other than the foreseeable AEs occurring within the first 7 days, or any AEs occurring after the first 7 days after vaccination.

9.9.1 Group holding rules

For safety reasons the first volunteer to receive a new vaccine dose in Groups 1-3 will be vaccinated alone and we will wait 48 hours before vaccinating subsequent volunteers. Two further volunteers may be vaccinated 48 hours after the first, and then at least another 48 hours gap will be left before vaccinating the rest of the volunteers receiving the same dose of the vaccine.

- **Solicited local adverse events:**
    - If 2 or more vaccinations in a group are followed by the same Grade 3 solicited local adverse event beginning within 2 days after vaccination (day of vaccination and one subsequent day) and persisting at Grade 3 for >48 hrs.

- **Solicited systemic adverse events:**
    - If 2 or more vaccinations in a group are followed by the same Grade 3 solicited systemic adverse event beginning within 2 days after vaccination (day of vaccination and one subsequent day) and persisting at Grade 3 for >48 hrs.

- **Unsolicited adverse events:**
    - If 2 or more vaccinations in a group are followed by the same Grade 3 unsolicited adverse event (including the same laboratory adverse event) that is considered possibly, probably or definitely related to vaccination and persists at Grade 3 for > 48hrs.

©Oxford University

- **A serious adverse event considered possibly, probably or definitely related to vaccination occurs**

- **Death occurs**

- **A life-threatening reaction occurs**

If a holding rule has been met we will inform the regulatory authority; following an internal safety review, if it is deemed appropriate to restart dosing, a request to restart dosing with pertinent data must be submitted to the regulatory authority as a request for a substantial amendment. The internal safety review will consider:

- The relationship of the AE or SAE to the vaccine.
- The relationship of the AE or SAE to the vaccine dose, or other possible causes of the event.
- If appropriate, additional screening or laboratory testing for other volunteers to identify those who may develop similar symptoms and alterations to the current Participant Information Sheet (PIS) are discussed.
- New, relevant safety information from ongoing research programs on the various components of the vaccine.

The sponsor, local ethics committee and vaccine manufacturers will also be notified if a holding rule is activated or released.

As per section 6.3.5, if a volunteer has an acute illness (moderate or severe illness with or without fever) or a fever (oral temperature greater than 37.5°C) at the scheduled time of administration of investigational product, the volunteer will not receive the vaccine at that time. The vaccine may be administered to that volunteer at a later date within the time window specified in the protocol (see Table 5) or they may be withdrawn from the study at the discretion of the Investigator.

All vaccinated volunteers will be followed for safety until the end of their planned participation in the study or until resolution or stabilisation (if determined to be chronic sequelae) of their AEs, providing they consent to this.

In addition to these pre-defined criteria, the study can be put on hold upon advice of the Local Safety Monitor, Chief Investigator, Study Sponsor, Regulatory Authority, Ethical Committee(s) or Local Safety Committee, for any single event or combination of multiple events which, in their professional opinion, jeopardise the safety of the volunteers or the reliability of the data.

©Oxford University

10. STATISTICS

This is a descriptive safety study, where volunteers will be vaccinated with a single dose of ChAdOx1 Chik. Twenty-four volunteers will be vaccinated in total. This sample size should allow an estimation to be made of the frequency and magnitude of outcome measures, rather than aiming to obtain statistical significance for differences between groups. Safety data will be presented according to frequency, severity and duration of adverse events.

The primary analysis for immunogenicity will be to assess the difference in magnitude of CHIKV specific T-cell and antibody responses between the groups. We will assess vaccine immunogenicity by comparing the change in these immunological parameters from baseline to different time points.

Statistical analysis will be conducted according to local SOPs and an agreed Statistical Analysis Plan when required.

Sample Size Selection

This is a descriptive phase I first in human trial that will balance the safety of volunteers with the aims to assess the vaccine's safety profile and immunogenicity after selected doses of the vaccines. The primary dose comparison will be between Groups 1, 2 and 3, which will have 6-9 subjects each. CHIKV specific immunogenicity will be the key immunological readout assessed by a variety of immunological assays.

©Oxford University

11. DATA MANAGEMENT

11.1 Data Handling

The Chief Investigator will be responsible for all data that accrues from the study. The data will be entered into the volunteers' CRFs in a paper and/or electronic format (using OpenClinica™ database). Electronic data will be stored on secure servers which are outsourced by OpenClinica™. Data will be entered in a web browser on PCs in the CCVTM building and then transferred to the OpenClinica Database by encrypted (Https) transfer. OpenClinica™ meets FDA part 11B standards. This includes safety data, laboratory data (both clinical and immunological) and outcome data.

Adverse event data will also be entered onto electronic or paper diaries by the volunteer

11.2 Record Keeping

The Investigators will maintain appropriate medical and research records for this trial, in compliance with GCP and regulatory and institutional requirements for the protection of confidentiality of volunteers. The Chief Investigator, co-Investigators and clinical research nurses will have access to records. The Investigators will permit authorised representatives of the Sponsor(s), as well as ethical and regulatory agencies to examine (and when required by applicable law, to copy) clinical records for the purposes of quality assurance reviews, audits and evaluation of the study safety and progress.

11.3 Source Data and Case Report Forms (CRFs)

All protocol-required information will be collected in CRFs designed by the Investigator. All source documents will be filed in the CRF. Source documents are original documents, data, and records from which the volunteer's CRF data are obtained. For this study, these will include, but are not limited to, volunteer consent form, blood results, GP response letters, laboratory records, diaries, and correspondence. In the majority of cases, CRF entries will be considered source data as the CRF is the site of the original recording (i.e. there is no other written or electronic record of data). In this study this will include, but is not limited to medical history, medication records, vital signs, physical examination records, urine assessments, blood results, adverse event data and details of vaccinations. All source data and volunteer CRFs will be stored securely.

11.4 Data Protection

The study protocol, documentation, data and all other information generated will be held in strict confidence. No information concerning the study or the data will be released to any unauthorised third party, without prior written approval of the sponsor.

©Oxford University

11.5 Data Quality

Data collection tools will undergo appropriate validation to ensure that data is collected accurately and completely. Datasets provided for analysis will be subject to quality control processes to ensure analysed data is a true reflection of the source data.

Trial data will be managed in compliance with local data management SOPs (including the overarching SOP OVC007 Data and Database Management). If additional, study specific information is required, an approved Data Management Plan will be implemented.

©Oxford University

12. QUALITY CONTROL AND QUALITY ASSURANCE PROCEDURES

12.1 Investigator procedures

Approved site-specific standard operating procedures (SOPs) will be used at all clinical and laboratory sites.

12.2 Monitoring

Monitoring will be performed according to ICH GCP by Clinical Trials Research Governance (CTRG). Following written SOPs, the monitors will verify that the clinical trial is conducted and data are generated, documented and reported in compliance with the protocol, GCP and the applicable regulatory requirements. The Investigator sites will provide direct access to all trial related source data/documents and reports for the purpose of monitoring and auditing by the Sponsor and inspection by local and regulatory authorities.

12.3 Protocol deviation

Any deviations from the protocol will be documented in a protocol deviation form and filed in the trial master file. Each deviation will be assessed as to its impact on volunteer safety and study conduct. Significant deviations will be listed in the end of study report.

12.4 Audit & inspection

The QA manager conducts systems based internal audits to check that trials are being conducted according to local procedures and in compliance with GCP and applicable regulations.

The Sponsor, trial sites, and ethical committee(s) may carry out audit to ensure compliance with the protocol, GCP and appropriate regulations.

GCP inspections may also be undertaken by the MHRA to ensure compliance with protocol and the Medicines for Human Use (Clinical Trials) Regulations 2004, as amended. The Sponsor will assist in any inspections and will support the response to the MHRA as part of the inspection procedure.

©Oxford University

13. SERIOUS BREACHES

The Medicines for Human Use (Clinical Trials) Regulations contain a requirement for the notification of "serious breaches" to the MHRA within 7 days of the Sponsor becoming aware of the breach.

A serious breach is defined as “A breach of GCP or the trial protocol which is likely to effect to a significant degree

(a) the safety or physical or mental integrity of the subjects of the trial; or

(b) the scientific value of the trial”.

In the event that a serious breach is suspected the Sponsor will be informed within one working day.

©Oxford University

14. ETHICS AND REGULATORY CONSIDERATIONS

14.1 Declaration of Helsinki

The Investigators will ensure that this study is conducted according to the principles of the current revision of the Declaration of Helsinki.

14.2 Guidelines for Good Clinical Practice

The Investigators will ensure that this study is conducted in full conformity with the Good Clinical Practice (GCP).

14.3 Approvals

The protocol, informed consent form, participant information sheet and any proposed advertising material will be submitted to an appropriate Research Ethics Committee (REC), HRA (where required), regulatory authorities (MHRA in the UK), and host institution(s) for written approval.

The Investigator will submit and, where necessary, obtain approval from the above parties for all substantial amendments to the original approved documents.

No substantial amendments to this protocol will be made without consultation with, and agreement of, the Sponsor. Any substantial amendments to the trial that appear necessary during the course of the trial must be discussed by the Investigator and Sponsor concurrently. If agreement is reached concerning the need for an amendment, it will be produced in writing by the Chief Investigator and will be made a formal part of the protocol following ethical and regulatory approval.

The Investigator is responsible for ensuring that changes to an approved trial, during the period for which regulatory and ethical committee(s) approval has already been given, are not initiated without regulatory and ethical committee(s)' review and approval except to eliminate apparent immediate hazards to the subject.

14.4 Volunteer Confidentiality

All data will be anonymised: volunteer data will be identified by a unique study number in the CRF and database. A separate confidential file containing identifiable information will be stored in a secured location in accordance with the Data Protection Act 1998. Only the Sponsor representative, Investigators, the clinical monitor, the REC and the MHRA will have access to the records. Photographs taken of vaccination sites (if required, with the volunteer's written, informed consent) will not include the volunteer's face and will be identified by the date, trial code and subject's unique identifier. Once developed, photographs will be stored as confidential records, as above. This material may be shown to

©Oxford University

other professional staff, used for educational purposes, or included in a scientific publication.

©Oxford University

15. FINANCING AND INSURANCE

15.1 Financing

The study is funded by Innovate UK.

15.2 Insurance

The University has a specialist insurance policy in place which would operate in the event of any participant suffering harm as a result of their involvement in the research (Newline Underwriting Management Ltd, at Lloyd's of London).

15.3 Compensation

Volunteers will be compensated for their time and for the inconvenience caused by procedures. They will be compensated £25 for attending the screening visit. For all other trial visits as outlined in Table 5, compensation will be calculated according to the following:

- Travel expenses:
    - £15 per visit. Where travel expenses are greater than £15 per visit because the volunteer lives outside the city of the trial site, the volunteer will be given further reimbursement to meet the cost of travel necessary for study visits.

- Inconvenience of blood tests:
    - £10 per blood donation

- Time required for visit:
    - £20 per hour

The total amount compensated will be approximately £370 depending on the exact number of visits and whether any repeat or additional visits are necessary.

©Oxford University

16. PUBLICATION POLICY

The Investigators will be involved in reviewing drafts of the manuscripts, abstracts, press releases and any other publications arising from the study. Data from the study may also be used as part of a thesis for a PhD or MD.

©Oxford University

17. REFERENCES

1. Suhrbier A, Jaffar-Bandjee MC, Gasque P. Arthritogenic alphaviruses--an overview. Nature reviews Rheumatology. 2012;8(7):420-9.

2. CDC. Chikungunya Virus Home - Geographic Distribution Centers for Disease Control and Prevention: Centers for Disease Control and Prevention; 2016 [Geographic distribution of Chikungunya virus]. Available from: https://www.cdc.gov/chikungunya/geo/index.html.

3. WHO. Chikungunya: Fact Sheet. 2016.

4. Schwartz O, Albert ML. Biology and pathogenesis of chikungunya virus. Nature reviews Microbiology. 2010;8(7):491-500.

5. Soumahoro MK, Boelle PY, Gauzere BA, Atsou K, Pelat C, Lambert B, et al. The Chikungunya epidemic on La Reunion Island in 2005-2006: a cost-of-illness study. PLoS neglected tropical diseases. 2011;5(6):e1197.

6. Burt FJ, Rolph MS, Rulli NE, Mahalingam S, Heise MT. Chikungunya: a re-emerging virus. Lancet (London, England). 2012;379(9816):662-71.

7. Pialoux G, Gauzere BA, Jaureguiberry S, Strobel M. Chikungunya, an epidemic arbovirosis. The Lancet Infectious diseases. 2007;7(5):319-27.

8. Weaver SC, Lecuit M. Chikungunya virus and the global spread of a mosquito-borne disease. The New England journal of medicine. 2015;372(13):1231-9.

9. Horstick O, Runge-Ranzinger S, Nathan MB, Kroeger A. Dengue vector-control services: how do they work? A systematic literature review and country case studies. Transactions of the Royal Society of Tropical Medicine and Hygiene. 2010;104(6):379-86.

10. Abdelnabi R, Neyts J, Delang L. Towards antivirals against chikungunya virus. Antiviral Research. 2015;121:59-68.

11. McSweegan E, Weaver SC, Lecuit M, Frieman M, Morrison TE, Hrynkow S. The Global Virus Network: Challenging chikungunya. Antiviral research. 2015;120:147-52.

12. Weaver SC, Osorio JE, Livengood JA, Chen R, Stinchcomb DT. Chikungunya virus and prospects for a vaccine. Expert review of vaccines. 2012;11(9):1087-101.

13. Erasmus JH, Rossi SL, Weaver SC. Development of Vaccines for Chikungunya Fever. The Journal of infectious diseases. 2016;214(suppl 5):S488-s96.

14. Modjarrad K. MERS-CoV vaccine candidates in development: The current landscape. Vaccine. 2016;34(26):2982-7.

15. Ramsauer K, Schwameis M, Firbas C, Müllner M, Putnak RJ, Thomas SJ, et al. Immunogenicity, safety, and tolerability of a recombinant measles-virus-based chikungunya vaccine: a randomised, double-blind, placebo-controlled, active-comparator, first-in-man trial. The Lancet Infectious Diseases. 2015;15(5):519-27.

16. Chang L-J, Dowd KA, Mendoza FH, Saunders JG, Sitar S, Plummer SH, et al. Safety and tolerability of chikungunya virus-like particle vaccine in healthy adults: a phase 1 dose-escalation trial. The Lancet. 2014;384(9959):2046-52.

17. Metz SW, Pijlman GP. Function of Chikungunya Virus Structural Proteins. In: Okeoma CM, editor. Chikungunya Virus: Advances in Biology, Pathogenesis, and Treatment. Cham: Springer International Publishing; 2016. p. 63-74.

18. Bett AJ, Haddara W, Prevec L, Graham FL. An efficient and flexible system for construction of adenovirus vectors with insertions or deletions in early regions 1 and 3. Proceedings of the National Academy of Sciences of the United States of America. 1994;91(19):8802-6.

19. Top FH, Jr., Grossman RA, Bartelloni PJ, Segal HE, Dudding BA, Russell PK, et al. Immunization with live types 7 and 4 adenovirus vaccines. I. Safety, infectivity, antigenicity, and

©Oxford University

potency of adenovirus type 7 vaccine in humans. The Journal of infectious diseases. 1971;124(2):148-54.

20. Grabenstein JD, Pittman PR, Greenwood JT, Engler RJ. Immunization to protect the US Armed Forces: heritage, current practice, and prospects. Epidemiol Rev. 2006;28:3-26.

21. Farina SF, Gao GP, Xiang ZQ, Rux JJ, Burnett RM, Alvira MR, et al. Replication-defective vector based on a chimpanzee adenovirus. J Virol. 2001;75(23):11603-13.

22. Bruna-Romero O, Gonzalez-Aseguinolaza G, Hafalla JC, Tsuji M, Nussenzweig RS. Complete, long-lasting protection against malaria of mice primed and boosted with two distinct viral vectors expressing the same plasmodial antigen. Proceedings of the National Academy of Sciences of the United States of America. 2001;98(20):11491-6.

23. Tatsis N, Tesema L, Robinson ER, Giles-Davis W, McCoy K, Gao GP, et al. Chimpanzee-origin adenovirus vectors as vaccine carriers. Gene Ther. 2006;13(5):421-9.

24. Dicks MD, Spencer AJ, Edwards NJ, Wadell G, Bojang K, Gilbert SC, et al. A novel chimpanzee adenovirus vector with low human seroprevalence: improved systems for vector derivation and comparative immunogenicity. PloS one. 2012;7(7):e40385.

25. Brandler S, Ruffié C, Combredet C, Brault J-B, Najburg V, Prevost M-C, et al. A recombinant measles vaccine expressing chikungunya virus-like particles is strongly immunogenic and protects mice from lethal challenge with chikungunya virus. Vaccine. 2013;31(36):3718-25.

26. Antrobus RD, Coughlan L, Berthoud TK, Dicks MD, Hill AV, Lambe T, et al. Clinical assessment of a novel recombinant simian adenovirus ChAdOx1 as a vectored vaccine expressing conserved Influenza A antigens. Mol Ther. 2014;22(3):668-74.

27. Antrobus RD, Berthoud TK, Mullarkey CE, Hoschler K, Coughlan L, Zambon M, et al. Coadministration of seasonal influenza vaccine and MVA-NP+M1 simultaneously achieves potent humoral and cell-mediated responses. Mol Ther. 2014;22(1):233-8.

28. Antrobus RD, Lillie PJ, Berthoud TK, Spencer AJ, McLaren JE, Ladell K, et al. A T cell-inducing influenza vaccine for the elderly: safety and immunogenicity of MVA-NP+M1 in adults aged over 50 years. PloS one. 2012;7(10):e48322.

29. Berthoud TK, Hamill M, Lillie PJ, Hwenda L, Collins KA, Ewer KJ, et al. Potent CD8+ T-cell immunogenicity in humans of a novel heterosubtypic influenza A vaccine, MVA-NP+M1. Clin Infect Dis. 2011;52(1):1-7.

30. Brady OJ, Gething PW, Bhatt S, Messina JP, Brownstein JS, Hoen AG, et al. Refining the global spatial limits of dengue virus transmission by evidence-based consensus. PLoS neglected tropical diseases. 2012;6(8):e1760.

31. Ewer KJ, O'Hara GA, Duncan CJ, Collins KA, Sheehy SH, Reyes-Sandoval A, et al. Protective CD8+ T-cell immunity to human malaria induced by chimpanzee adenovirus-MVA immunisation. Nature communications. 2013;4:2836.

32. Borthwick N, Ahmed T, Ondondo B, Hayes P, Rose A, Ebrahimsa U, et al. Vaccine-elicited human T cells recognizing conserved protein regions inhibit HIV-1. Molecular therapy : the journal of the American Society of Gene Therapy. 2014;22(2):464-75.

33. Swadling L, Capone S, Antrobus RD, Brown A, Richardson R, Newell EW, et al. A human vaccine strategy based on chimpanzee adenoviral and MVA vectors that primes, boosts, and sustains functional HCV-specific T cell memory. Sci Transl Med. 2014;6(261):261ra153.

34. Green CA, Scarselli E, Sande CJ, Thompson AJ, de Lara CM, Taylor KS, et al. Chimpanzee adenovirus- and MVA-vectored respiratory syncytial virus vaccine is safe and immunogenic in adults. Science translational medicine. 2015;7(300):300ra126.

35. Ewer K, Rampling T, Venkatraman N, Bowyer G, Wright D, Lambe T, et al. A Monovalent Chimpanzee Adenovirus Ebola Vaccine Boosted with MVA. The New England journal of medicine. 2016;374(17):1635-46.

©Oxford University
