# Supplementary material for: A single dose of ChAdOx1 Chik vaccine induces neutralizing antibodies against four chikungunya virus lineages in a phase 1 clinical trial
Source: Nat Commun. 2021 Jul 30;12:4636. doi: 10.1038/s41467-021-24906-y (PMC8324904; doi:10.1038/s41467-021-24906-y)
Supplement: Supplementary file 3 — Reporting Summary [file 41467_2021_24906_MOESM3_ESM.pdf]

nature research

Corresponding author(s): Arturo Reyes-Sandoval

Last updated by author(s): Jun 24, 2021

Reporting Summary

Nature Research wishes to improve the reproducibility of the work that we publish. This form provides structure for consistency and transparency in reporting. For further information on Nature Research policies, see our Editorial Policies and the Editorial Policy Checklist.

Statistics

For all statistical analyses, confirm that the following items are present in the figure legend, table legend, main text, or Methods section.

n/a	Confirmed	
☐	☒	The exact sample size (*n*) for each experimental group/condition, given as a discrete number and unit of measurement
☐	☒	A statement on whether measurements were taken from distinct samples or whether the same sample was measured repeatedly
☐	☒	The statistical test(s) used AND whether they are one- or two-sided<br>*Only common tests should be described solely by name; describe more complex techniques in the Methods section.*
☒	☐	A description of all covariates tested
☐	☒	A description of any assumptions or corrections, such as tests of normality and adjustment for multiple comparisons
☐	☒	A full description of the statistical parameters including central tendency (e.g. means) or other basic estimates (e.g. regression coefficient) AND variation (e.g. standard deviation) or associated estimates of uncertainty (e.g. confidence intervals)
☐	☒	For null hypothesis testing, the test statistic (e.g. *F*, *t*, *r*) with confidence intervals, effect sizes, degrees of freedom and *P* value noted<br>*Give P values as exact values whenever suitable.*
☒	☐	For Bayesian analysis, information on the choice of priors and Markov chain Monte Carlo settings
☒	☐	For hierarchical and complex designs, identification of the appropriate level for tests and full reporting of outcomes
☒	☐	Estimates of effect sizes (e.g. Cohen's *d*, Pearson's *r*), indicating how they were calculated

Our web collection on statistics for biologists contains articles on many of the points above.

Software and code

Policy information about availability of computer code

Data collection	OpenClinica (Enterprise Edition) v3.13
Data analysis	Prism v 9.1.0 (GraphPad), BioTek Gen5 v3.09, FlowJo v10.6.2 (BD Biosciences), Clustal Omega (https://www.ebi.ac.uk/Tools/msa/clustalo/).

For manuscripts utilizing custom algorithms or software that are central to the research but not yet described in published literature, software must be made available to editors and reviewers. We strongly encourage code deposition in a community repository (e.g. GitHub). See the Nature Research guidelines for submitting code & software for further information.

Data

Policy information about availability of data

All manuscripts must include a data availability statement. This statement should provide the following information, where applicable:

- Accession codes, unique identifiers, or web links for publicly available datasets
- A list of figures that have associated raw data
- A description of any restrictions on data availability

There is a restriction on the availability of the data presented on this manuscript due to the data being used to feed a patent application and because data will be linked to an ongoing Phase 1b blinded study funded by a different research award. Anonymised participant data may be available upon requests directed to the corresponding author (arturo.reyes@ndm.ox.ac.uk). Proposals will be reviewed and approved by the sponsor (CTRG- https://researchsupport.admin.ox.ac.uk/ctrg#/), principal investigator, and collaborators on the basis of scientific merit. If approved and upon signature of a data access agreement, data can be shared through a secure online platform. Data sharing may take a period of up to 6 weeks from receiving the request. All data will be made available for a minimum of 5 years from the end of the trial. The study protocol is available with this publication as part of the supplementary material.

Field-specific reporting

Please select the one below that is the best fit for your research. If you are not sure, read the appropriate sections before making your selection.

[x] Life sciences [ ] Behavioural & social sciences [ ] Ecological, evolutionary & environmental sciences

For a reference copy of the document with all sections, see nature.com/documents/nr-reporting-summary-flat.pdf

Life sciences study design

All studies must disclose on these points even when the disclosure is negative.

Sample size

The sample size was selected based on other previous phase I trials using the same vector. This sample size was able to detect significant differences in immune responses from baseline while exposing a limited number of people to an investigational medicinal product that was being used for the first time. The number of recruited participants allows an estimation to be made of the frequency and magnitude of outcome measures, rather than aiming to obtain statistical significance for differences between groups.

Data exclusions

All available data from eligible volunteers was included in the manuscript, with the exception of 1 individual data point within the PRNT data. The data point from 1 participant in the low dose group was excluded from PRNT analysis due to failure in QC.

Replication

* Since this a first-in-human clinical trial study, it was not possible to perform further biological replicates as to the number of participants recruited within this trial. A phase 2 clinical trial will inform and validate the results from this phase 1 study.

* ELISpot assays- Assays were ran on fresh cells, therefore samples could not be repeated. Responses were average across 3 technical triplicates and the mean response of the unstimulated (negative control) wells were subtracted. QC criteria were applied to samples before inclusion and analysis. For a sample to be considered valid, at least two technical replicates had to pass the pre-established QC parameters. We did not have any data point failing QC. Plates were counted using an AID automated ELISpot counter (AID Diagnostika GmbH, algorithm C) using identical calibration settings for all plates. Spot counts were adjusted only to remove artifacts.

* PRNT assays- Were initially ran as a single replicate for each biological sample, but validated by re-running around 30-40% of the samples in a second experiment. Internal positive and negative controls were included. Counting of plaques was performed by a single blinded operator.

* Intracellular cytokine staining for flow cytometry- Experiments were ran on fresh cells, as a single replicate for each biological sample. QC criteria were applied to samples before inclusion and analysis. To maintain reproducibility between sample batches, the same cytometer and the same lot of antibodies were used for every run. Gating and analysis was performed by a single operator. We did not have any data point failing QC.

* ELISA assays - Were ran as 3 technical replicates for each biological sample. Each of the 96-well plates contained: a standard curve in duplicate, blank wells in triplicate and an internal control in triplicates. The internal control was prepared as an independent dilution to correspond to the 4th serial dilution of the standard curve. The standard curve OD values at 450 nm were fitted to a four-parameter model using the BioTek Gen5 v3.09 software. QC criteria were applied to the curve to ensure that the test samples were interpolated similarly between plates and that the coefficient of variance was low (<20%). Failure to meet these parameters resulted in failure of the assay and the whole sample plate was repeated. We did not have any data point failing QC.

Randomization

This study was not randomized because required a dose-escalation approach and the IMP was not compared against placebo.
To assess immune responses, each participant was compared to its own baseline prior to vaccination.

Blinding

There was not blinding between the participants and the clinical team due to the type of study design (one product at dose-escalation). Scientists performing PRNT assays were blinded to the volunteer vaccination dosage group and time point. It was not possible to blind the ELISpot and ICS sample processing due to the fact that the samples had to be processed in fresh. Independent scientists from those performing the assays performed QC of all immunogenicity assays.

Reporting for specific materials, systems and methods

We require information from authors about some types of materials, experimental systems and methods used in many studies. Here, indicate whether each material, system or method listed is relevant to your study. If you are not sure if a list item applies to your research, read the appropriate section before selecting a response.

Materials & experimental systems

n/a	Involved in the study
	☒ Antibodies
	☒ Eukaryotic cell lines
☒	☐ Palaeontology and archaeology
☒	☐ Animals and other organisms
	☒ Human research participants
	☒ Clinical data
☒	☐ Dual use research of concern

Methods

n/a	Involved in the study
☒	☐ ChIP-seq
	☒ Flow cytometry
☒	☐ MRI-based neuroimaging

Antibodies

Antibodies used

ELISpot:
Anti-human IFN-γ, capture IgG1 mouse monoclonal Ab, clone 1-D1K (MABTECH, cat# 3420-3) - dilution 1:100.
Anti-human IFN-γ biotinylated , detection mouse IgG1 monoclonal Ab, clone 7-B6-1 (MABTECH, cat# 3420-6) - dilution 1:1000.

ELISA:
Anti-human IgG (γ-chain specific)-Alkaline phosphatase polyclonal antibody produced in goat (Sigma-Aldrich, cat# A3187).

Flow cytometry:
Anti-human CD14 eFluor450, clone HIB19, mouse IgG1monoclonal Ab (eBioscience UK, cat# 48-0199-42) - dilution 1:100.
Anti-human CD19 eFluor450, clone 61D3, mouse IgG1 monoclonal Ab (eBioscience UK, cat# 48-0149-42) - dilution 1:100.
Anti-human CD3 AF700, clone UCHT1, mouse IgG1monoclonal Ab (eBioscience UK, cat# 56-0038-82) - dilution 1:50.
Anti-human CD4 APC, clone RPA-T4, mouse IgG1 monoclonal Ab (eBioscience UK, cat# 17-0049-42) - dilution 1:25.
Anti-human CD8a APC eFluor780, clone RPA-T8, mouse IgG1 monoclonal Ab (eBioscience UK, cat# 47-0088-42) - dilution 1:10.
Anti-human IFN-γ FITC, clone 4S.B3, mouse IgG1 monoclonal Ab (eBioscience UK, cat# 11-7319-82) - dilution 1:250.
Anti-human TNF-a PE-Cy7, clone MAb11, mouse IgG1 monoclonal Ab (eBioscience UK, cat# 25-7349-82)- dilution 1:500.
Anti-human IL-2 PE, clone MQ1-17H12, rat IgG2a monoclonal Ab (eBioscience UK, cat# 12-7029-82) - dilution 1:50.
Anti-human CD28 , clone CD28.2, functional grade mouse IgG1 monoclonal Ab (eBioscience UK, cat# 16-0289-85) - used at 1 μg/mL
Anti-human CD49d (Integrin alpha 4), clone 9F10, functional grade mouse IgG1 monoclonal Ab(eBioscience UK, cat# 16-0499-85) - used at 1 μg/mL

Validation

ELISpot:
The use of the same lot number ensured consistency within the assay and over time.
• Anti-human IFN-γ monoclonal Ab, clone 1-DIK (MABTECH). The manufacturer confirms on its website that this is antibody has been purposely designed to be used in ELISpot (https://www.mabtech.com/products/anti-human-ifn-gamma-antibody-1-d1k-purified-3420-3)
• Anti-human IFN-γ biotinylated monoclonal Ab, clone 7-B6-1 (MABTECH). The manufacturer confirms on its website that this is antibody has been purposely designed to be used in ELISpot (https://www.mabtech.com/products/anti-human-ifn-gamma-antibody-7-b6-1-biotinylated-3420-6)

ELISA:
The use of the same lot number ensured consistency within the assay and over time.
• Anti-human IgG (γ-chain specific)-Alkaline phosphatase polyclonal antibody produced in goat (Sigma-Aldrich, cat# A3187). The manufacturer confirms on its website that this is antibody has been purposely designed to be used in ELISA (https://www.sigmaaldrich.com/catalog/product/sigma/a3187?lang=en&region=GB)

Flow cytometry:
Each primary antibody was titrated in-house to determine optimal concentration. The use of the same lot number ensured consistency within the assay and over time.
• Anti-human CD14 eFluor450, clone HIB19- The manufacturer confirms on its website that this antibody has been purposely designed to be used in flow cytometry (https://www.thermofisher.com/antibody/product/CD14-Antibody-clone-61D3-Monoclonal/48-0149-42)
• Anti-human CD19 eFluor450, clone 61D3- The manufacturer confirms on its website that this antibody has been purposely designed to be used in flow cytometry (https://www.thermofisher.com/antibody/product/CD14-Antibody-clone-61D3-Monoclonal/48-0149-42)
• Anti-human CD3 AF700, clone UCHT1- The manufacturer confirms on its website that this antibody has been purposely designed to be used in flow cytometry and that it was verified by Relative expression to ensure that the antibody binds to the antigen stated (https://www.thermofisher.com/antibody/product/CD3-Antibody-clone-UCHT1-Monoclonal/56-0038-82)
• Anti-human CD4 APC, clone RPA-T4- The manufacturer confirms on its website that this antibody has been purposely designed to be used in flow cytometry (https://www.thermofisher.com/antibody/product/CD4-Antibody-clone-RPA-T4-Monoclonal/17-0049-42)
• Anti-human CD8a APC eFluor780, clone RPA-T8 - The manufacturer confirms on its website that this antibody has been purposely designed to be used in flow cytometry (https://www.thermofisher.com/antibody/product/CD8a-Antibody-clone-RPA-T8-Monoclonal/47-0088-42)
• Anti-human IFN-γ FITC, clone 4S.B3- The manufacturer confirms on its website that this antibody has been purposely designed to be used in flow cytometry and that it was verified by Relative expression to ensure that the antibody binds to the antigen stated

(https://www.thermofisher.com/antibody/product/IFN-gamma-Antibody-clone-4S-B3-Monoclonal/11-7319-82)
• Anti-human TNF-a PE-Cy7, clone MAb11 - The manufacturer confirms on its website that this antibody has been purposely designed to be used in flow cytometry (https://www.thermofisher.com/antibody/product/TNF-alpha-Antibody-clone-MAb11-Monoclonal/25-7349-82)
• Anti-human IL-2 PE, clone MQ1-17H12 - The manufacturer confirms on its website that this antibody has been purposely designed to be used in flow cytometry and that it was verified by Relative expression to ensure that the antibody binds to the antigen stated (https://www.thermofisher.com/antibody/product/IL-2-Antibody-clone-MQ1-17H12-Monoclonal/12-7029-82).
• Anti-human CD28 , clone CD28.2 - The manufacturer confirms on its website that this antibody has been reported for use in flow cytomety and for costimulation of T cells in in-vitro functional assays (https://www.thermofisher.com/antibody/product/CD28-Antibody-clone-CD28-2-Monoclonal/16-0289-85).
• Anti-human CD49d (Integrin alpha 4), clone 9F10- The manufacturer confirms on its website that this antibody has been reported for use in flow cytomety and in in-vitro functional assays (https://www.thermofisher.com/antibody/product/CD49d-Integrin-alpha-4-Antibody-clone-9F10-Monoclonal/16-0499-85)

Eukaryotic cell lines

Policy information about cell lines

Cell line source(s)	Vero Cells CCL-81 were purchased from ATCC (https://www.atcc.org/products/ccl-81)
Authentication	The provider offered a certificate of analysis when purchased.
Mycoplasma contamination	Cells lines tested negative for mycoplasma contamination.
Commonly misidentified lines (See ICLAC register)	None.

Human research participants

Policy information about studies involving human research participants

Population characteristics	24 healthy adult UK residents, between 18 and 45years old, were recruited.<br>Table 1 on this manuscript describes the baseline characteristics of the recruited participants.
Recruitment	Volunteers were recruited by use of advertisement formally approved by the ethics committees. Advertisement was distributed around the city of Oxford or posted on-line (University website and social media). Before any participant became enrolled, a formal in-person screening visit took place. During this visit all study procedures were explained, inclusion and exclusion criteria were assessed and informed consent was obtained.<br>Volunteers were excluded from the study if they were concurrently involved in another trial. In order to check this, volunteers were asked to provide their National Insurance or Passport number (if they are not entitled to a NI number) and were registered on a national database of participants in clinical trials (www.tops.org.uk).<br>There is a potential for self selection bias, but this is a recognised limitation of first-in-human studies where we are purposely selecting healthy adults rather than trying to obtain a sample that is representative of the general population. This is why we have planned further phase 1 / 2 studies with larger sample sizes.
Ethics oversight	This study was approved within the UK by the Medicines and Healthcare Products Regulatory Agency (MHRA reference 21584/0394/001-0001) and the South Central Oxford A Research Ethics Committee (REC reference 18/SC/0004).

Note that full information on the approval of the study protocol must also be provided in the manuscript.

Clinical data

Policy information about clinical studies

All manuscripts should comply with the ICMJEguidelines for publication of clinical research and a completedCONSORT checklist must be included with all submissions.

Clinical trial registration	NCT03590392
Study protocol	The study protocol is available with this publication as part of the supplementary material.
Data collection	The study took place at a single site in the UK (Centre for Clinical Vaccinology and Tropical Medicine, University of Oxford).<br>Participants' screenings and recruitment began in August 2018, with last participant's visit on 30th September 2019.
Outcomes	Study objectives and outcomes are described within the study protocol.<br><br>Primary Outcome Measures:<br>The specific endpoints for safety and reactogenicity were actively and passively collected data on adverse events. The following parameters were assessed by:<br>* Occurrence of solicited local reactogenicity signs and symptoms for 7 days following the vaccination.<br>* Occurrence of solicited systemic reactogenicity signs and symptoms for 7 days following the vaccination.<br>* Occurrence of unsolicited adverse events for 28 days following the vaccination.<br>* Change from baseline for safety laboratory measures.

* Occurrence of serious adverse events during the whole study duration.

Secondary Outcome Measures:
To assess the cellular and humoral immunogenicity. The following parameters were assessed by:
* ELISA to quantify antibodies to CHIKV E2 protein.
* Ex vivo ELISpot responses to CHIKV protein antigens.

Exploratory immunology:
* PRNT50 to assess cross-neutralizing antibodies against different CHIKV isolates.
* Flow cytomeytry to evaluate cytokine responses by main T cell populations.

Flow Cytometry

Plots

Confirm that:

- [x] The axis labels state the marker and fluorochrome used (e.g. CD4-FITC).
- [x] The axis scales are clearly visible. Include numbers along axes only for bottom left plot of group (a 'group' is an analysis of identical markers).
- [x] All plots are contour plots with outliers or pseudocolor plots.
- [x] A numerical value for number of cells or percentage (with statistics) is provided.

Methodology

Sample preparation

Intracellular cytokine staining (ICS) was performed on freshly isolated PBMC from peripheral venous blood. PBMC were resuspended in R10 media (RPMI+10% HIFBS) containing anti-human CD49d and anti-human CD28. PBMC were then placed in 5mL polypropylene FACS tubes and stimulated (at 37oC, 5% CO2) with pools of overlapping peptides spanning the structural polyprotein of CHIKV (see supplementary information for a description of the used peptides). Brefeldin A and monensin were added after 2 hours and incubation continued overnight for 16-20 hours.
At the end of the incubation cells were washed with FACS buffer and stained with a viability dye (Aqua Live/dead, Life Technologies Ltd) for 20 minutes at room temperature in the dark. Cells were washed with FACS buffer and fixed with Cytofix/Cytoperm buffer (Becton Dickinson) for 20 minutes at room temperature in the dark. A wash with permeabilisation wash buffer (Becton Dickinson) was performed before proceeding with staining. An intracellular staining cocktail was prepared containing the flow cytometry antibodies listed in the section above. The cocktail was added to the cells and incubated for 30 min at room temperature in the dark. Finally cells were washed with permeabilisation wash buffer and resuspended in FACS buffer. Samples were stored at 4oC and acquired within 24 hours from staining.

Instrument

LSRFortessa - 4 lasers (Becton Dickinson)

Software

FACSDiva v 8.02 (BD Biosciences).
FlowJo v10.6.2 (BD Biosciences).
Prism v 9.1.0 (GraphPad).

Cell population abundance

The cell population of interest was single viable CD3+ lymphocytes. The minimum number of viable CD3+ cells recorded was 400,000 and the maximum was 900,000.

Gating strategy

Lymphocytes (FSC-A vs. SSC-A) < Single cells (FSC-A vs. FSC-H) < CD14/CD19- (SSC-A vs CD19-CD14) < Live CD3+ (Aqua Live/dead vs. CD3)
From the live CD3+ cells, two populations were gated as follows:
* CD4+ T cells were obtained after selection of CD4+ and subsequent exclusion of CD8+
* CD8+ T cells were obtained after selection of CD8+ and subsequent exclusion of CD4+
For both CD4+ and CD8+ populations, we gated for the following cytokines:
* IFN-g+ and subsequent boolean gating for TNF-a+ and IL-2.
* TNF-a+ and subsequent boolean gating for IFN-g+ and IL-2.
* IL-2+ and subsequent boolean gating for IFN-g+ and TNF-a.

- [x] Tick this box to confirm that a figure exemplifying the gating strategy is provided in the Supplementary Information.
